# Mathematical Modeling Identifies Inhibitors of Apoptosis as Mediators of Positive Feedback and Bistability

**DOI:** 10.1371/journal.pcbi.0020120

**Published:** 2006-09-15

**Authors:** Stefan Legewie, Nils Blüthgen, Hanspeter Herzel

**Affiliations:** Institute for Theoretical Biology, Humboldt University, Berlin, Germany; Memorial Sloan Kettering Cancer Center, United States of America

## Abstract

The intrinsic, or mitochondrial, pathway of caspase activation is essential for apoptosis induction by various stimuli including cytotoxic stress. It depends on the cellular context, whether cytochrome c released from mitochondria induces caspase activation gradually or in an all-or-none fashion, and whether caspase activation irreversibly commits cells to apoptosis. By analyzing a quantitative kinetic model, we show that inhibition of caspase-3 (Casp3) and Casp9 by inhibitors of apoptosis (IAPs) results in an implicit positive feedback, since cleaved Casp3 augments its own activation by sequestering IAPs away from Casp9. We demonstrate that this positive feedback brings about bistability (i.e., all-or-none behaviour), and that it cooperates with Casp3-mediated feedback cleavage of Casp9 to generate irreversibility in caspase activation. Our calculations also unravel how cell-specific protein expression brings about the observed qualitative differences in caspase activation (gradual versus all-or-none and reversible versus irreversible). Finally, known regulators of the pathway are shown to efficiently shift the apoptotic threshold stimulus, suggesting that the bistable caspase cascade computes multiple inputs into an all-or-none caspase output. As cellular inhibitory proteins (e.g., IAPs) frequently inhibit consecutive intermediates in cellular signaling cascades (e.g., Casp3 and Casp9), the feedback mechanism described in this paper is likely to be a widespread principle on how cells achieve ultrasensitivity, bistability, and irreversibility.

## Synopsis

Multicellular organisms eliminate damaged or excess cells by programmed cell death, also known as apoptosis. By modelling the signaling pathways involved in the initiation of apoptosis, the authors provide insight into how cells prevent spontaneous apoptosis, but yet efficiently enter cell death, once proapoptotic signals exceed a threshold. The simulations also explain how cells accurately translate a complex set of pro- and anti-apoptotic signals into a life-or-death decision. Once apoptosis has been initiated, cellular demise must irreversibly proceed even if the initial trigger is removed, as partial cellular disintegration might lead to tissue inflammation or cellular deregulation. The authors explain how such irreversible commitment arises in the initiation pathways of apoptosis and provide experimentally testable predictions. Finally, the simulations reveal an unanticipated role for the inhibitor of apoptosis family of proteins, as these proteins are predicted to be involved in the amplification of death signals and not only in their suppression.

## Introduction

Apoptosis, an evolutionary conserved form of cell suicide, allows multicellular organisms to eliminate damaged or excess cells in order to maintain tissue homeostasis. Dysregulation of apoptosis is associated with various pathological conditions, including cancer and neurodegenerative disorders. Aspartate-specific cysteine proteases, also known as caspases, are the central executioners of apoptosis. In most cases, apoptotic stimuli activate initiator caspases, whose substrates, the effector caspases, ultimatively cause cellular demise by cleaving various cellular substrates [1].

**Figure 1:**
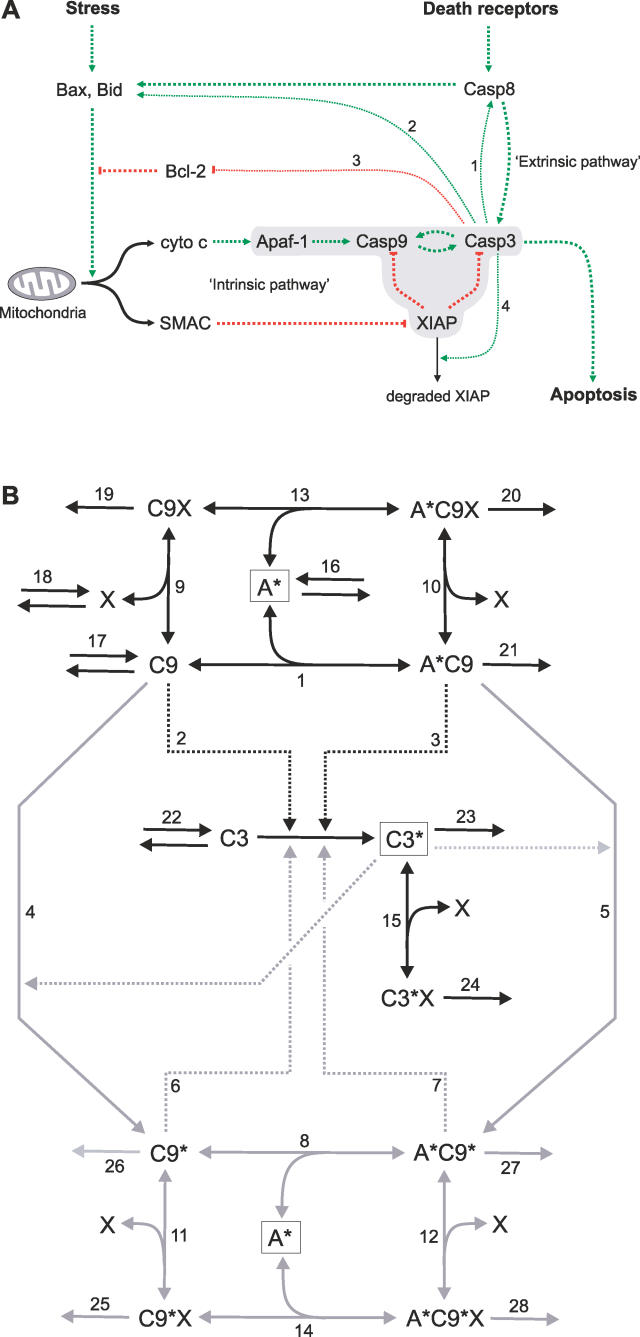
Mathematical Model of the Intrinsic Apoptosis Pathway (A) Schematic representation of intrinsic and extrinsic apoptosis pathways. Dotted lines indicate positive (green) or negative (red) regulation, and the solid lines refer to release of Smac and cyto c from mitochondria. The regulatory interactions considered in the model are highlighted in gray. The numbers 1–4 refer to additional feedbacks described in the Discussion. (B) Kinetic scheme of the model. The reactions depicted in gray, which are involved in Casp3-mediated feedback cleavage of Casp9, were eliminated in the Casp9-mutant model in order to dissect the role of XIAP-mediated feedback. A*, activated Apaf-1; C3, Casp3; C9, Casp9.

Figure 1A schematically depicts the so-called extrinsic and intrinsic apoptotic pathways that elicit apoptosis by cleaving and thereby activating caspase-3 (Casp3), the major cellular effector caspase. The extrinsic pathway is initiated by ligand-binding to death receptors (e.g., CD95), which then oligomerize and recruit various proteins, including pro-Casp8, into the so-called death-inducing signaling complex. Formation of the death-inducing signaling complex leads to autoprocessing of pro-Casp8 into active (initiator) Casp8, which then cleaves (effector) Casp3. Cytotoxic stress or death-receptor–stimulated Casp8 engage the intrinsic, or mitochondrial, apoptosis pathway by inducing the translocation of proapoptotic Bcl-2 family members such as Bax and Bid to mitochondria. This event, which is negatively regulated by antiapoptotic Bcl-2 family members (e.g., Bcl-2), results in the release of proapoptotic proteins (cytochrome c [cyto c] and Smac) from mitochondria into the cytosol. Cytosolic cyto c then elicits the oligomerization of Apaf-1 into an active high-molecular-weight complex, the apoptosome, which recruits and stimulates (initiator) Casp9, and thereby allows activation of effector caspases such as Casp3. Smac and inhibitors of apoptosis (IAPs) such as X-linked IAP (XIAP) establish an additional layer of regulation in the intrinsic pathway: XIAP inhibits the catalytic activities of Casp9 and Casp3 through reversible binding, and cytosolic Smac relieves this inhibition by sequestering XIAP away from caspases [2].

Experimental studies revealed that the qualitative behaviour of caspase activation in the intrinsic pathway depends on the cellular context. Cyto c added to cytosolic extracts activates Casp3 in an all-or-none fashion in some cells [3–7], while gradual activation was observed in other systems [8–10]. As cyto-c release from mitochondria can be a reversible event [11], which does not affect mitochondrial function [12–14], it has been suggested that downstream caspase activation irreversibly commits cells to apoptosis [15,16]. Accordingly, cyto c–induced Casp3 activation remained elevated even after a strong decline in cytosolic cyto c [17] or after apo–cyto c, an inhibitor of apoptosome formation, was added [18]. Furthermore, the time course of caspase activation via the intrinsic pathway equals that for irreversible commitment to apoptosis [15,16], and caspase-inhibition allows for long-term cellular recovery and/or proliferation after removal of apoptotic stimuli [15,16,19–22]. Finally, Fas-treated Jurkat T cells, which enter apoptosis by the intrinsic pathway, escaped commitment to death as judged by maintenance of clonogenic potential if Casp3 was inhibited [23]. On the contrary, Casp3 activation was found to be a reversible event in glycochenodeoxycholate-treated hepatocytes [24].

These qualitative differences in caspase activation suggest that the intrinsic pathway is bistable in some cells, but monostable in others. While simple monostable systems respond in a gradual and reversible manner, complex bistable systems exhibit true all-or-none responses and in some cases irreversibility. Bistability is thought to require a positive circuit, which may be established either by positive feedback or by double-negative feedback. Once a threshold stimulus is exceeded, such positive circuits allow bistable systems to switch from low activation levels (off state) to high activation levels (on state) in an all-or-none fashion. Bistable systems display hysteresis, meaning that different stimulus-response curves are obtained depending upon whether the system began in its off or its on state. In some cases, the on state is maintained indefinitely after the stimulus is removed, so that the system shows irreversible activation [25]. Experimental studies confirmed that bistability indeed occurs in natural and artificial biological networks [25–29].

Recent mathematical modeling demonstrated that bistability can arise from “hidden,” or implicit, feedback loops that are usually not explicitly drawn in biochemical reaction schemes [30,31]. Similarily, we present a model showing that inhibition of Casp3 and Casp9 by IAPs results in an implicit positive feedback and in bistability. As cellular inhibitory proteins (e.g., IAPs) frequently inhibit consecutive intermediates in cellular signaling cascades (e.g., Casp3 and Casp9), the mechanism described in this paper is likely to be a widespread principle on how cells achieve ultrasensitivity, bistability, and irreversibility (Protocol S5).

## Results

### Model Derivation

Based on the published literature, we derived a core model of the intrinsic apoptosis pathway, which includes general regulatory mechanisms, while cell-type–specific events were not taken into account. The gray-shaded area in Figure 1A indicates the regulatory interactions considered in the model: active Apaf-1, which was taken as the input in most simulations, recruits and thereby stimulates (initiator) Casp9. Casp9 then in turn activates the output species, (effector) Casp3, by proteolytic processing. In addition, Casp3-mediated cleavage of Casp9 results in positive feedback amplification. Finally, both Casp3 and Casp9 are subject to stoichiometric inhibition by IAPs. For simplicity, we considered only the most potent caspase inhibitor among the IAP family of proteins, XIAP. The corresponding kinetic scheme is depicted in Figure 1B.

Cyto c released from mitochondria is known to elicit heptamerization of Apaf-1 into active apoptosomes. As detailed kinetic measurements of apoptosome formation are currently lacking, apoptotic stimulation was modeled by altering the total concentration of activated Apaf-1 molecules assembled in apoptosomes (A*_tot_ = A* + A*C9 + A*C9X + A*C9* + A*C9*X). Each active Apaf-1 monomer assembled in apoptosomes was shown to reversibly bind to a single Casp9 molecule [32], and Casp9 is then autoproteolytically processed at amino acid Asp-315 [33]. Importantly, Casp9 autoproteolysis does not affect either enzymatic activity of Casp9 [34] or its recruitment to apoptosomes [35,36]. Because of these data, we did not distinguish between autoproteolytically processed and unprocessed Casp9 in the model.

The enzymatic activity of Casp9 is thought to be determined mainly by apoptosome recruitment, as apoptosome-bound Casp9 was shown to be much more active than free Casp9 [37,38]. Therefore, we assumed in the model that reversible association of Casp9 (C9) and Apaf-1 (A*) (reaction 1) yields a highly active Apaf1–Casp9 complex (A*C9), which cleaves pro-Casp3 (C3) much more efficiently (reaction 3) than free Casp9 (reaction 2; see Table 1). The latter reaction was nevertheless taken into account, since free Casp9 was shown to have significant basal activity towards pro-Casp3 [32].

**Table 1:**
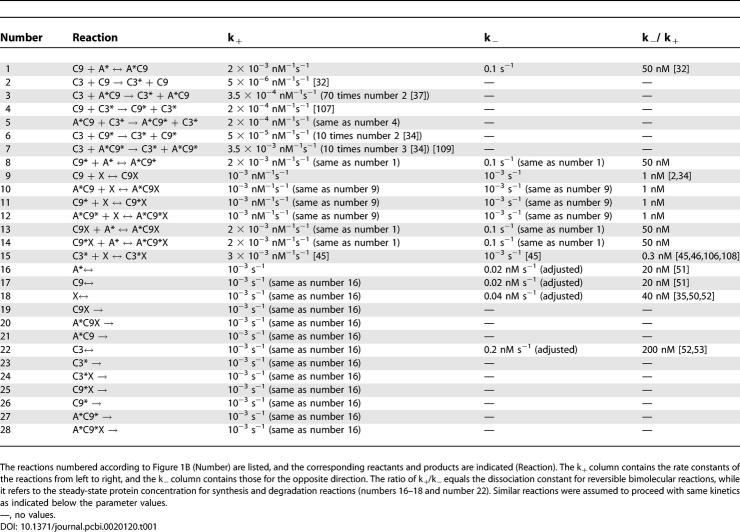
Kinetic Parameters

Processing of pro-Casp3 into mature Casp3 by upstream initiator caspases such as Casp9 was reported to occur by a sequential two-step mechanism: Pro-Casp3, which has negligible enzymatic activity [39], is initially processed by Casp9 into active p12-p20-Casp3, and this intermediate is subsequently autocatalytically cleaved into active p12-p17-Casp3 [40]. As shown in Figure 1B, we modeled Casp3 activation by a single-step mechanism (C3 → C3*). This seems justified, as the p12-p20-Casp3 intermediate and mature p12-p17-Casp3 exhibit similar catalytic activities [41], and as they are both subject to inhibition by XIAP (see below).

Casp3 is known to cleave its own activator, Casp9, at amino acid Asp-330 in vitro [34,42], and in cytosolic extracts treated with cyto c [5,33]. As Casp9 processing by Casp3 was shown to significantly enhance Casp9 activity [34], feedback cleavage by Casp3 results in autoamplification of the apoptotic signal. The physiological relevance of this positive feedback loop was confirmed in several studies, which showed that inhibition of Casp3-mediated cleavage of Casp9 prevented full activation of both Casp3 and Casp9 in response to cyto c [34,43,44].

Casp3-mediated feedback processing of Casp9 was modeled by assuming that active Casp3 (C3*) cleaves both free and Apaf1-associated Casp9 (reactions 4 and 5), thereby generating the Asp330-cleaved Casp9 species, C9* and A*C9*. These feedback-cleaved Casp9 species in turn cleave pro-Casp3 more efficiently (reactions 6 and 7; see Table 1) when compared with their precursors, C9 and A*C9*, thus establishing a feedback amplification loop. Feedback-processed Casp9 (cleaved at Asp330) was shown to be associated with apoptosomes [34,36], much like its precursors that are not cleaved at Asp330 (see above). Therefore, we assumed in the model that the kinetics of Casp9-binding to Apaf-1 (reactions 1 and 8) are unaffected by Casp3-mediated feedback cleavage (see Table 1).

IAPs such as XIAP act as stoichiometric inhibitors of Casp3 and Casp9 [2], and accordingly caspase inhibition can be described by simple reversible binding [45,46]. Experimental evidence suggests that XIAP can bind to and inhibit Casp9, even if the latter is associated with apoptosomes [34]. Accordingly, we assumed in the model that active Apaf-1 (A*) and XIAP (X) bind to Casp9 in a noncompetitive manner so that Apaf1-bound Casp9 intermediates (A*C9 and A*C9*) recruit XIAP with the same kinetics as free Casp9 (C9 and C9*). In addition, we modeled XIAP binding to Casp9 such that it is not affected by either Casp9 autocleavage (at Asp-315) or Casp3-mediated feedback cleavage (at Asp-330). As contradictory experimental results were obtained on how Casp9 cleavage modulates inhibition by XIAP, the impact of the latter assumption will be stressed in the Discussion.

Because of the assumptions made in the previous paragraph, there is reversible recruitment of XIAP to all Casp9 species in the model (reactions 9–12), and also free exchange of Apaf1 between the resulting Casp9–XIAP complexes (reactions 13–14). All Casp9–XIAP complexes were assumed to be catalytically inactive, which is in accordance with experimental studies [47,48]. Furthermore, Casp3-mediated feedback processing of XIAP-bound Casp9 was neglected in the model, as the Casp9–XIAP binding interface is nearby the corresponding cleavage site (Asp-330) [48].

It is well established that XIAP binds both to partially processed Casp3 (p12-p20) and to mature Casp3 (p12-p17), but not to its inactive precursor pro-Casp3 [46,49]. In accordance with experimental data [45,46], reversible association between Casp3 and XIAP (reaction 15) was modeled to result in a catalytically inactive complex (C3*X). Due to the enzymatic inactivity of pro-Casp3 (C3) [39] and of the Casp3–XIAP complex (C3*X), free active Casp3 (C3*) was taken as the response in our simulations.

Finally, we included protein synthesis and degradation in the model (reactions 16–28). More specifically, the unmodified proteins A*, C9, X, and C3 are produced with a constant rate, and all molecular species in Figure 1B are subject to first-order degradation. While the total cellular concentrations of Apaf-1, Casp9, Casp3, and XIAP (i.e., the ratio of protein synthesis and degradation rates) were measured [35,50–53], the kinetics of synthesis and degradation were not known. For simplicity, we assumed the same degradation rate for all molecular species in the model, and adjusted the synthesis rates in order to obtain previously measured protein concentrations (Table 1). This implies that the total concentrations of Apaf-1, Casp9, Casp3, and XIAP remained constant throughout our simulations.

From the model described above (Figure 1B), which will be referred to as the “wild-type model” in the following, molecular balances could be derived for each considered molecular species resulting in a system of 13 ordinary differential equations (Protocol S1). In general, protein–protein association (reactions 1, 4–6, 7, and 10–13 in Figure 1B) was modeled as a reversible second-order process, and caspase-mediated cleavage (reactions 2, 3, 8, 9, 14, and 15 in Figure 1B) was modeled as an irreversible second-order process. As many similar reactions (e.g., 1 and 13 in Figure 1B) were assumed to proceed with the same kinetics (see Table 1), the model comprises 16 kinetic parameters. The unknown kinetic parameters were set to reasonable values (Table 1) in order to reproduce the previously reported time courses of caspase activation (see “Time Course of Casp3 Activation”).

Besides the wild-type model, we also analyzed two modified models in order to get insights into the mechanisms that are responsible for bistability in caspase activation. 1) In the “Casp9-mutant model,” which comprises *only* the black reactions in Figure 1B, we eliminated Casp3-mediated feedback cleavage of Casp9 (reactions 8 and 9 in Figure 1B) from the wild-type model. 2) Based on available experimental data (see Discussion), we assumed competitive (i.e., mutually exclusive) binding of Casp3 and Casp9 to XIAP in the wild-type model. By contrast, Casp3 and Casp9 were allowed to bind XIAP simultaneously in the “noncompetitive model”; that is, the wild-type model was extended by four ternary Casp9-XIAP-Casp3 complexes (Protocol S1).

### Time Course of Casp3 Activation

Experiments in cytosolic extracts revealed that exogenously added cyto c induces maximal Casp3 cleavage within ~15 min in some cells [36], while completion takes longer (up to ~60 min) in other systems [5,6,33,54]. More specifically, the Casp3 cleavage seems to be fast upon strong stimulation, but slower if stimulation is weak [6,53,55].

We were interested whether the model was able reproduce these observations if previously measured protein concentrations of Apaf-1 (20 nM), Casp9 (20 nM), Casp3 (200 nM), and XIAP (40 nM) were assumed [35,50–53]. Exogenous addition of cyto c was simulated by a step-like increase in the total amount of active Apaf-1 monomers, A*_tot_, as cyto c–induced apoptosome formation was reported to be a very rapid process [36,38]. Such a step-input is also expected to reflect input characteristics within living cells reasonably well, since cyto c release from mitochondria was shown to complete within 5 min [56,57].

**Figure 2:**
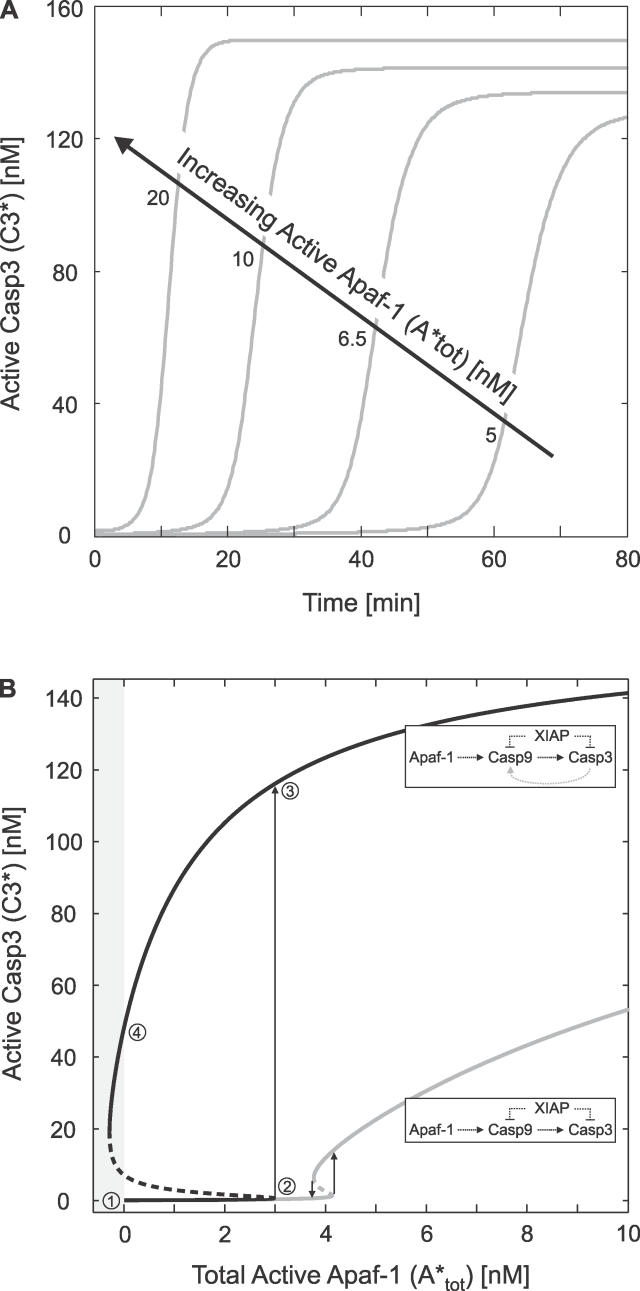
Dynamic and Steady-State Behaviour of the Caspase Cascade (A) Time course of Casp3 activation (wild-type model) upon a step-like increase in the amount of active Apaf-1 (A*_tot_) at t = 0 from zero to the concentration indicated. (B) Steady-state stimulus-response curves of the wild-type model (black line) and of the Casp9-mutant model (gray line), where Casp3-mediated feedback cleavage of Casp9 does not occur. Stable and unstable steady states are indicated by solid and dashed lines, respectively.

The results shown in Figure 2A reveal that the simulated time courses of caspase activation agree well with those measured experimentally, and that simulated response time is indeed inversely related to the stimulus strength (Figure 2A). Full activation of all cellular Apaf-1 molecules (A*_tot_ = 20 nM) elicits fast Casp3 activation, while a critical slowing down is observed near the threshold (A*_tot_ ~ 3 nM; see Figure 2B), as expected for a bistable system [28,50]. Notably, the slope of the time courses shown in Figure 2A is only marginally affected by the onset time of caspase activation (i.e., by the stimulus level). This is in accordance with experimental results obtained in cyto c–treated cytosolic extracts [5,36,54] and in single living cells [58], which showed that, once initiated, Casp3 activation is rapidly completed within less than 15 min.

### Bistability in Caspase Activation

Experimental evidence suggests that cyto c–induced caspase activation can be bistable and irreversible (see Introduction). The simulated steady-state Casp3 activity (C3*) was indeed bistable and irreversible (Figure 2B, black line). The system exhibits three steady states, two stable (solid black lines) and one unstable (dashed black line), for A*_tot_ between 0 and ~3 nM, and shows hysteretic behaviour: starting from the resting state (point 1), the system retains low Casp3 activity even for increasing stimuli, A*_tot_, until a threshold (point 2) is reached, whereby Casp3 activity switches to the higher steady state (point 3) in an all-or-none fashion. The system remains at this higher steady state even if the stimulus is removed (point 4), so that caspase activation is irreversible, and thus represents the point of no return for apoptosis.

We next addressed the mechanism of bistability, and hypothesized that Casp3-mediated feedback cleavage of Casp9 was responsible, since bistability is thought to require a positive circuit [25]. Therefore, reactions 8 and 9 in Figure 1B were blocked to simulate a mutant Casp9 (D330A), which is refractory to cleavage by Casp3 (“Casp9-mutant model”). Unexpectedly, bistability was retained (Figure 2B, gray line), which suggests that a hidden positive feedback loop operates in the Casp9-mutant model.

### XIAP Establishes an Implicit Positive Feedback in Caspase Activation

**Figure 3:**
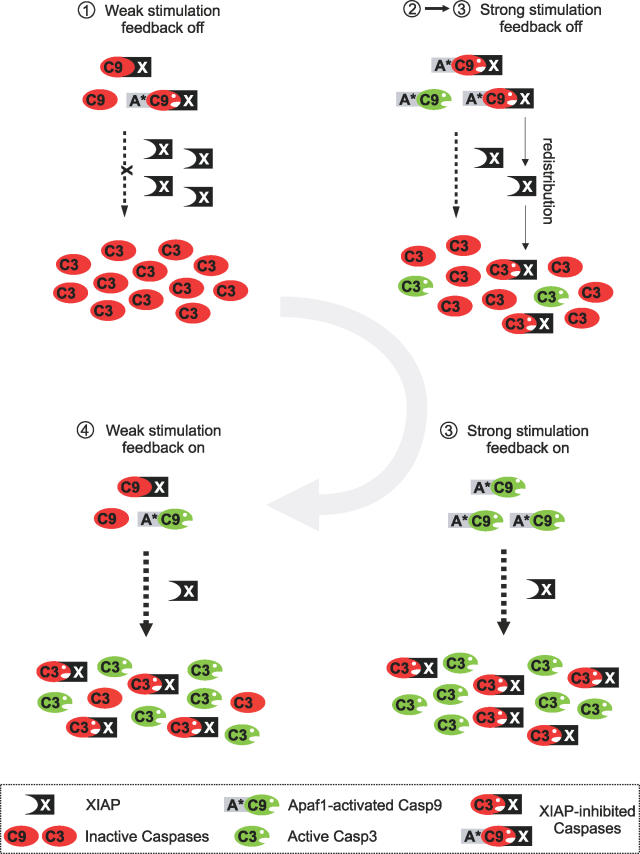
Schematic Representation of XIAP-Mediated Feedback At resting state (top left) Casp9 is efficiently inhibited by XIAP, so that Casp3 is inactive. Upon stronger stimulation (top right) some Casp9 escapes XIAP-mediated inhibition and activates Casp3, which then sequesters XIAP away from Casp9 (redistribution). This XIAP redistribution finally results in strong activation of both Casp9 and Casp3 (bottom right), and retains the system in an active state even if the stimulus is reduced (bottom left). The numbers on the top of each scheme correspond to those indicated next to the stimulus-response in Figure 2B (black line).

More detailed simulations revealed that XIAP establishes an implicit positive feedback in the Casp9-mutant model, and Figure 3 schematically depicts how this mechanism contributes to irreversibility in the wild-type model: Upon weak stimulation (point 1 in Figure 2B) the vast majority of Apaf1-associated, highly active Casp9 molecules is inhibited by excess XIAP, so that cleavage of pro-Casp3 is negligible (top left in Figure 3). As the stimulus strength is increased above the threshold (point 2 in Figure 2B), active Apaf-1 also recruits some free Casp9 that is not subject to inhibition by XIAP, so that Casp3 activation is initiated (top right in Figure 3). Active Casp3 then further promotes its own activation by sequestering XIAP away from Apaf-1-associated Casp9 (“redistribution”), so that finally the vast majority of XIAP is bound to Casp3 (bottom right in Figure 3). This XIAP redistribution results a positive feedback loop, which, together with Casp3-mediated Casp9 feedback cleavage, suddenly switches the system from low to high Casp3 activity (transition from point 2 to point 3 in Figure 2B). Caspase activity is maintained even if the stimulus is removed, as Casp3, once activated, retains XIAP, and thereby prevents full Casp9 deactivation (bottom left in Figure 3). Additional simulations, which corroborate our conclusions regarding XIAP-mediated feedback can be found in Protocol S2.

**Figure 4:**
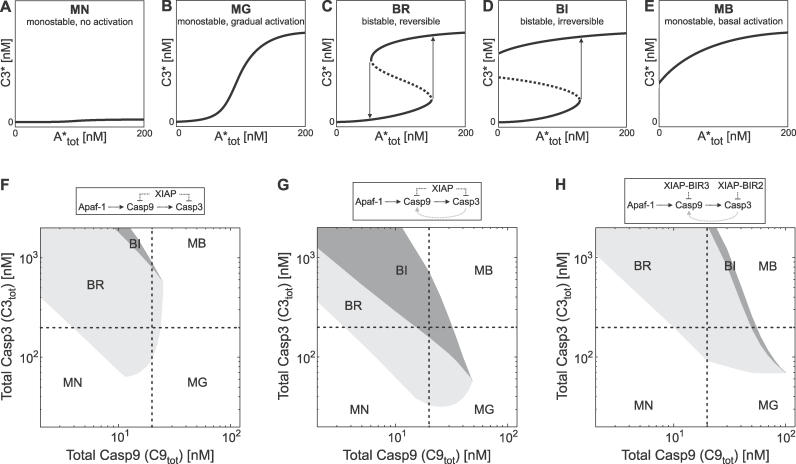
Determinants for Bistability and Irreversibility I The dose-response curves of the Casp9-mutant model (F), those of the wild-type model (G), and those obtained for noncompetitive caspase binding to XIAP (H) were analyzed for varying Casp3 and Casp9 expression levels. Five types of qualitative behaviour, which are schematically depicted in (A–E), could be distinguished in the physiological range of Apaf-1 expression levels (0–200 nM). The light and dark gray areas in (F–H) correspond to the bistable regions of the model (BR, BI), and the abbreviations MN, MG, and MB indicate the qualitative behaviour outside the bistable region. Experimentally measured caspase concentrations (see Table 1) are highlighted by dashed lines in (F–H).

In order to determine how the protein concentrations in the caspase cascade affect bistability, we analyzed the stimulus–response curves (similar to those in Figure 2) for varying total Casp3 and Casp9 concentrations. Five types of qualitative behaviour in caspase activation could be distinguished in the physiological range of stimulus concentrations (A*_tot_ = 0–200 nM): 1) the system is essentially devoid of any Casp3 activation (monostable–no activation [MN], Figure 4A); 2) Casp3 activation occurs in a gradual manner (monostable–gradual activation [MG], Figure 4B); 3) the caspase cascade is bistable-reversible (BR, Figure 4C); 4) Casp3 activation is bistable-irreversible (BI, Figure 4D); and 5) constitutive Casp3 activity is observed (monostable–basal activation [MB], Figure 4E). The corresponding bifurcation diagram (Figure 4F) reveals that bistability in the Casp9 mutant model can only be observed if the total Casp9 concentration is lower than that of XIAP (40 nM), which ensures that the system is in the off-state as long as Casp3 is inactive. In addition, Casp3 must be significantly more abundant than XIAP to sequester it away from Casp9 (i.e., to establish positive feedback).

### Determinants for Bistability and Irreversibility

We next sought to determine the relative contribution of XIAP-mediated feedback and that of Casp3-mediated feedback cleavage (of Casp9) to bistability and irreversibility in caspase activation. To this end, we compared the bifurcation plot of the wild-type model (Figure 4G) with those of mutant models, where we selectively blocked either XIAP-mediated feedback (Figure 4H; “noncompetitive model”) or Casp3-mediated feedback cleavage (Figure 4F, “Casp9-mutant model”). XIAP-mediated feedback is abolished in the noncompetitive model (Protocol S1), since XIAP was assumed to be capable of simultaneous binding to Casp3 and Casp9 in these simulations. As schematically depicted above Figure 4H, this corresponds to a caspase cascade, which is controlled by the XIAP fragments BIR1-BIR2 (specific for Casp3) and BIR3-RING (specific for Casp9) rather than by full-length XIAP. Figure 4F and 4H demonstrate that each feedback mechanism alone can bring about bistability for experimentally measured caspase expression levels (interception of dashed lines in Figure 4F–4H). By contrast, irreversibility is restricted to a narrow range of caspase concentrations in both mutant models, and is never observed in the vicinity of experimentally measured caspase expression levels. Importantly, the wild-type model exhibits robust irreversibility in the physiological range of caspase expression levels, which suggests that irreversibility in caspase activation requires coordinated action of both XIAP- and cleavage-mediated feedbacks.

The computational results shown in Figure 4G also explain why various cell types show qualitatively different patterns of caspase activation and unravel the underlying mechanisms: Casp3 activation is efficiently inhibited in cells, where the total XIAP concentration exceeds those of Casp3 and Casp9 (MN; Figure 4A). Gradual Casp3 activation is predicted to occur in cells, where Casp9 expression is high compared with XIAP and Casp3 expression (MG, Figure 4B). In this situation XIAP is effectively sequestered by excess Casp9, and the remaining free Casp9 molecules efficiently cleave Casp3 as if XIAP was not present. In case that both caspases are expressed at intermediate levels, the feedback loops discussed above cooperate to reversibly switch on the system in an all-or-none fashion (BR, Figure 4D). Even higher caspase expression levels relieve the cascade from XIAP-mediated inhibition so that Casp3 can be highly active even in the absence of stimulation. Such constitutive activation either arises spontaneously (MB, Figure 4C) or requires previous suprathreshold Casp3 activation (BI, Figure 4E).

**Figure 5:**
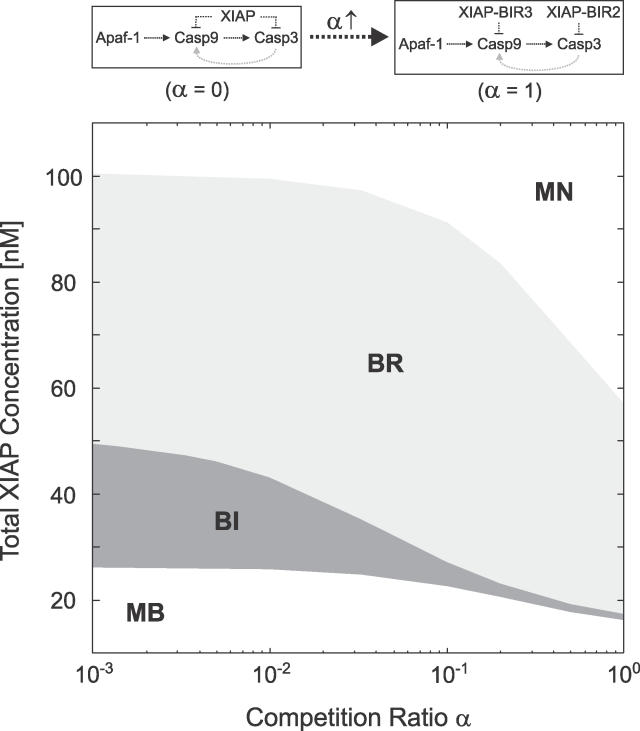
Determinants for Bistability and Irreversibility II The qualitative behaviour of caspase activation according to Figure 4A–4E is shown as a function of the XIAP level, and of the competition ratio α. The competition ratio α (Protocol S1) equals the fold-change in XIAP's affinity for Casp9 brought about by Casp3 binding to XIAP (and vice versa), and thereby quantifies the degree of competitive caspase binding to XIAP as indicated on the top.

The preceding conclusions could be confirmed by analyzing the qualitative behaviour of caspase activation as a function of the competition ratio α, and of XIAP expression (Figure 5). The competition ratio α equals the fold-change in XIAP's affinity for Casp9 brought about by Casp3 binding to XIAP (and vice versa), and thereby quantifies the degree of competitive caspase binding to XIAP (Protocol S1). Figure 5 demonstrates that the range of bistability is significantly broadened even if the Casp3-binding to XIAP reduces XIAP's affinity for Casp9 (and vice versa) less than 5-fold (α > 0.2). By contrast, reliable irreversibility requires significant competition of caspases for XIAP, at least with the default protein concentrations (Table 1) we assumed here. As shown in Figure 5, high XIAP levels completely abolish caspase activation (MN), bistability is observed for intermediate XIAP concentrations (BR, BI), and low XIAP levels fail to prevent caspase activation even in the absence of external stimulation (MB).

Our conclusions regarding the qualitative behaviour of caspase activation are supported by experimental data. 1) Overexpression of XIAP abolishes apoptosis and Casp3 activation in response to microinjection of cyto c (type MN) [59]. 2) overexpression of Casp3 [60,61] or Casp9 [33,62,63] results in caspase activation and/or apoptosis (type MB). In contrast, Casp3 overexpression failed to elicit its own activation in another study [64], and the model suggests that this may be due to low Casp9 expression (see Figure 4G). 3) High levels of IAP antagonists such as Smac were shown to activate the Casp9 → Casp3 pathway [65,66] and to elicit spontaneous apoptosis [67], even in cell types that are devoid of basal cyto c release or Casp8 activation (type MB). The inability of others to reproduce Casp3 activation by XIAP depletion or Smac addition [68] is probably due to the fact that the threshold BI → MB (Figure 5) was not exceeded in these studies (e.g., due to the expression of Smac-resistant IAP proteins such as NAIP [69]). 4) Gradual Casp3 activation (type MG) was observed in cyto c–treated cytosolic extracts [8–10], and also in flow cytometric analyses of living cells [70,71]. 5) The existence of bistable states (types BI and BR) is supported by all-or-none Casp3 activation in response to cyto c, and by the fact that Casp3 activation can irreversibly commit cells to death (see Introduction), although definitive proof for these types of behaviour is lacking (see Discussion).

### The Mitochondrial Pathway Acts as an Efficient Binary Integrator

In the previous section, we demonstrated that excess of XIAP over Casp3 and Casp9 abolishes cyto c–induced caspase activation even if high concentrations (200 nM) of the stimulus, active Apaf-1, were assumed (type MN). However, various experimental studies in cells, where Casp3 activation was inhibited downstream of cyto c release, have shown that caspase activation can be rescued by relatively moderate Apaf-1 overexpression (see [72] and references therein). This suggests that Casp3 activation does not occur if the concentration of the bottleneck, active Apaf-1, is below the threshold stimulus concentration, where the bistable system switches from the lower to the upper steady state (point 2 in Figure 2B). In support for such a threshold model, it was recently shown that a minor (~2-fold) decrease in Apaf-1 expression dramatically decreases caspase activation in response to cyto c microinjection [72]. These studies also suggest that the apoptotic threshold can be regulated downstream of Apaf-1, as Smac, an inhibitor of XIAP action, rescued cyto c–induced caspase activation in Apaf1-knockdown cells [72]. Therefore, we were interested how the threshold of the bistable cascade is affected by transcriptional and post-transcriptional regulation of Casp3, Casp9, and/or XIAP.

**Figure 6:**
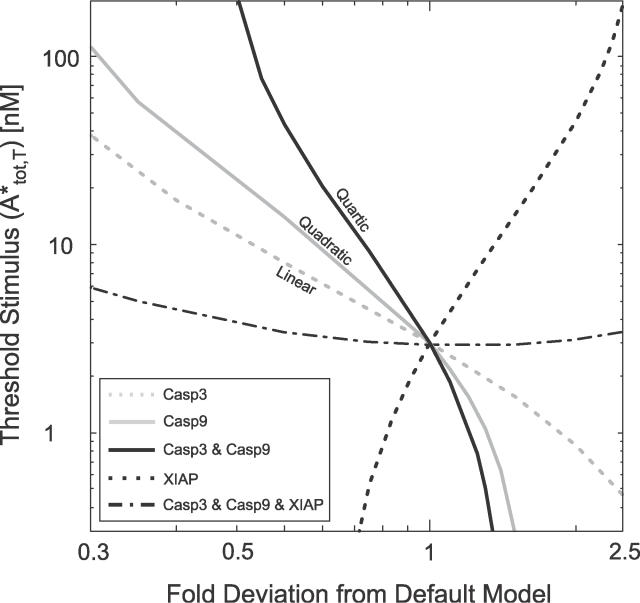
Binary Integration of Multiple Inputs The threshold stimulus, A*_tot,T_, where the bistable system switches from the lower to the higher steady state (point 2 in Figure 2B), is plotted as a function of Casp3 (gray dotted line), Casp9 (gray solid line), or XIAP (black dashed line) expression. In addition, the impact of simultaneous alterations of Casp3 *and* Casp9 (black solid line) or of Casp3, Casp9, *and* XIAP (black dash-dotted line) to the same relative extent is shown. The intersection of the graphs corresponds to the default protein concentrations (see Table 1). The terms “linear,” “quadratic,” and “quartic” indicate the relationship between protein expression and the apoptotic threshold, A*_tot,T_.

The corresponding results are shown in Figure 6: starting from the default model (point of intersection), the predicted threshold stimuli, A*_tot,T_, of the bistable system were plotted as a function of Casp3 (gray dotted line), Casp9 (gray solid line), and XIAP (black solid line) expressions. In addition, we also considered simultaneous alterations of Casp3 and Casp9 to the same relative extent (black solid line) in order to understand how the apoptotic threshold is affected by nitric oxide, a covalent inhibitor of Casp3 and Casp9 active sites [1]. These simulations demonstrate that decreasing levels of Casp3 moderately increase the threshold, A*_tot,T_, while alterations in Casp9 shift the threshold more efficiently. Regulation of XIAP levels is predicted to allow even more effective control over the apoptotic threshold, and similar arguments also hold for nitric oxide–mediated inhibition of both Casp3 *and* Casp9 [1].

Our results regarding XIAP as an efficient modulator of an all-or-none threshold are in accordance with experimental studies, as a 2-fold drop in XIAP expression was sufficient to allow cyto c–induced Casp3 activation [73,74]. Moreover, increasing amounts of Smac, a high-affinity inhibitor of XIAP (Figure 1B), elicited all-or-none Casp3 activation in cyto c–treated HeLa cell cytosols [68]. Finally, the threshold cyto c concentration that is required to achieve switch-like Casp3 activation was shown to be cell-type dependent, and low thresholds correlated with low IAP expression levels [7]. Our simulations are also corroborated by the fact that PKB/Akt-mediated inhibitory phosphorylation of Casp9 completely abolished cyto c–induced Casp3 activation, even though Casp9 enzymatic activity was only partially suppressed [63]. In addition, Casp3 overexpression sensitizes cells to apoptosis in response to cytotoxic stress [64], which is also in accordance with the simulations shown in Figure 6.

Thus, we can conclude that bistable behaviour in the mitochondrial caspase cascade serves to compute multiple regulatory inputs into a binary decision whether caspase activation occurs or not (“binary integrator”). Further calculations, where relative changes in protein expression were related to relative changes in the threshold stimulus, A*_tot_, suggest that the following order of input potency holds in general: Regulation of active Apaf-1 < Casp3 regulation < Casp9 regulation < XIAP regulation ≈ simultaneous regulation of Casp3 *and* Casp9. The simulations also predict that the apoptotic threshold is essentially constant if all components (i.e., Casp3, Casp9 *and* XIAP) are simultaneously changed to the same relative extent (Figure 6; black dash-dotted line). Hence, the life-or-death decision appears to be remarkably insensitive towards random fluctuations in gene expression, which are thought to result in correlated changes in cellular protein levels [75]. In addition, these simulations suggest that general inhibitors of protein synthesis or degradation, which are known to be inducers of apoptosis [56,64], do not affect the threshold of the Casp9 → Casp3 cascade.

## Discussion

In the present paper we showed that inhibition of Casp3 and Casp9 by IAPs results in an implicit positive feedback, since cleaved Casp3 augments its own activation by sequestering IAPs away from Casp9 (Figure 3). In addition, we demonstrated that XIAP-mediated feedback cooperates with Casp9 cleavage by Casp3 to bring about bistable and irreversible Casp3 activation in the range of experimentally measured kinetic parameters and protein concentrations (Figures 2, 4, and 5).

### Model Assumptions

XIAP-mediated feedback can only be observed if Casp3 and Casp9 compete for binding to XIAP at least to some extent (Figures 3 and 5). Such competition is supported by the fact that Casp3 and Casp9 cannot be co-immunoprecipitated in cells [35]. Casp3 (and not only Casp9) is recruited to the apoptosome at least in some cells [35], and it is conceivable that this occurs by means of a sequential Apaf1-Casp9-XIAP-Casp3 complex. Even if such a complex exists, it seems to be rather instable, as Casp3 can be eluted from the apoptosome (i.e., from Apaf-1) by low ionic strength [39], while much higher ionic strength is required to elute Casp9 [32]. Recent co-immunoprecipitation experiments revealed the existence of a ternary Casp9-XIAP-Casp3 complex in vitro [76]. However, only minor amounts of Casp3 were found in the complex even if XIAP was incubated with excess Casp3 and Casp9. Taken together, these data suggest that Casp3 and Casp9 significantly compete for binding to XIAP.

Co-immunoprecipitation studies with Casp3, Casp9, and XIAP might underestimate the degree of competition of caspases for a single XIAP molecule (i.e., XIAP-mediated feedback), as IAP family members are often homodimers. In case that each XIAP molecule in a dimer independently couples to caspases, a ternary Casp9–XIAP–Casp3 complex will be seen, even if Casp3 and Casp9 compete for a *single* XIAP molecule. Therefore, we propose to directly test for XIAP-mediated feedback in vitro. As further outlined in Protocol S3, a Casp9 mutant (D330A), which is refractory to Casp3-mediated feedback cleavage, should be incubated with active apoptosomes and XIAP either in the presence or in the absence of pro-Casp3. Coincubation with XIAP alone is expected to result in low Casp9 activity [34], but excess pro-Casp3 should reverse this inhibition by sequestering XIAP away from Casp9.

We have also assumed in the model that XIAP inhibits all forms of Casp9 (i.e., that the affinity between Casp9 and XIAP is not affected by either Casp9 autocleavage [at Asp-315] or Casp3-mediated feedback cleavage of Casp9 [at Asp-330]). While it is clear that autoprocessed Casp9 (cleaved at Asp-315 only) is efficiently inhibited by XIAP [34,47,49,51,77], some authors reported that XIAP also binds to and inhibits uncleaved pro-Casp9 [34,47,78], at least partially [77,79], but others could not reproduce these results [49,51]. As explained in the context of Figure 3, bistability requires that XIAP binds to and inhibits Apaf1-activated Casp9 upon weak stimulation, so that low Casp3 activity can be maintained. Importantly, such XIAP-mediated control over Casp9 activity will be ensured even if XIAP does not associate with uncleaved pro-Casp9, since pro-Casp9 recruitment to the apoptosome was shown to result in its fast and complete autoprocessing (at Asp-315) [38,39]. Casp3-mediated feedback cleavage (at Asp-330) was reported to relieve Casp9 from inhibition by XIAP [51], and might thereby establish an additional positive feedback, which would further broaden the ranges of bistability and irreversibility. As other experimental studies do not support the existence of this additional feedback [34,49], we have made the conservative assumption that XIAP inhibits feedback-cleaved Casp9, too.

In our core model of the intrinsic pathway we considered only Casp3 and XIAP, but not functionally redundant molecules. For example, Casp7, which is activated by Casp9 [5], also mediates XIAP-mediated feedback, since it efficiently binds to IAPs [46]. Likewise, molecules such as c-IAP1, c-IAP2, and NAIP are functionally redundant to XIAP, as they inhibit both Casp3 and Casp9 [2,69]. In case such functionally redundant proteins are expressed, the protein concentrations varied in our simulations (e.g., C3_tot_ in Figure 3F–3H) represent combinations (e.g., sums) of functionally redundant protein concentrations (e.g., C3_tot_ and C7_tot_), so that the results given in the paper continue to hold.

### Input Signals

We used the concentration of active Apaf-1 assembled into apoptosomes as the varying input signal in our simulations, rather than the amount of cyto c released from mitochondria. This seems justified, as available experimental evidence suggests that apoptosome formation increases gradually with increasing cyto c concentration [10,37], and that signal amplification occurs in the caspase cascade considered in this paper [10]. Our model explains how cells reject erroneous cyto c release from single mitochondria, and also predicts that reversible cyto c release can elicit irreversible caspase activation. It should be noted that cyto c release upon apoptotic stimulation was reported to be all or none under many [56,57] but not all [80,81] circumstances. Importantly, dose-response curves using active Apaf-1 as the input (e.g., Figure 2B) are physiologically relevant even if cyto c release is all or none, as they help to explain why caspase activation is completely abolished for limiting Apaf-1 expression (see [72] and references therein). More generally, the model provides insights into how the intrinsic pathway integrates multiple regulatory inputs including including cyto c release, cyto c sequestration [39], transcriptional regulation of Apaf-1 [72], Apaf-1 sequestration [39], transcriptional regulation of IAPs [2], Smac-mediated IAP sequestration [2], Casp9 phosphorylation [63], and caspase S-nitrosylation [1]. As shown in Figure 6, the caspase cascade acts as a binary integrator in the range of bistability (BI and BR in Figures 4 and 5). In contrast, gradual integration will be seen if the system resides in the monostable–gradual activation range, and this is particularly relevant for apoptotic stimuli that directly regulate caspase cascade members (e.g., Apaf-1) in addition to releasing cyto c (“feedforward regulation”). For example, p53 is known to induce Apaf-1 expression [82], and thereby can elicit gradual Casp3 activation even if cyto c release is all or none. Alternatively, gradual Casp3 activation, which was seen in flow cytometric analyses of living cells [70,71], may be due to cell-to-cell variability in the intrinsic pathway. Such cellular heterogeneity seems to be significant, as cyto c injection alone or in combination with Smac does not elicit Casp3 activation [83] or cell death [59,84] in all cells of a population. Our model provides a reasonable basis for further studies that focus on cell-to-cell variability in the intrinsic pathway.

In the Results section, we referred to experimental studies where Smac, a competitive, high-affinity inhibitor of IAP binding to caspases [85], was either added to cytosolic extracts or microinjected into living cells. In living cells, Smac is eventually released simultaneously with cyto c from mitochondria [2] (see Figure 1A). Importantly, such physiological release of Smac simply corresponds to decreasing XIAP levels in our model, as most experiments with caspase inhibitors have shown that Smac release does not require caspase-mediated feedback [57,86–88]. Thus, the results shown in Figure 6 explain why simultaneous release of cyto c and Smac is required to elicit Casp3 activation in many cell types (e.g., [83]), and predict that these two stimuli are integrated in an all-or-none manner.

### Upstream, Downstream, and Feedback Signaling

In accordance with previous experimental studies (see Introduction), we showed that, depending on the protein expression levels in the intrinsic pathway, caspase activation irreversibly commits cells to apoptosis (BI regions in Figures 4 and 5). However, some cells die by a delayed and morphologically distinct form of cell death, so-called caspase-independent cell death, even if caspases are inhibited [89]. Because caspase-independent cell death is thought to be initiated at the level of mitochondria, our simulations do not unravel the determinants for commitment to death in these cells, but only those for commitment to the fastest death pathway (i.e., apoptosis). As the precise kinetics of cell death may, for example, be important in development [89], our results are likely to be relevant even in cells subject to caspase-independent cell death. The physiological importance of the caspase cascade considered in our model is further supported by the fact that Apaf-1, Casp9, and Casp3 knockout mice show morphological defects and die early in development [89]. In addition, caspase inhibition (e.g., due to IAP overexpression) allowed for long-term cellular survival and mitochondrial recovery in response to cytotoxic stress [19–23] and/or after cyto c was released [11,13,15].

Other positive feedbacks than those included in the model have been described in the literature. For example, Casp3 was shown to induce processing of Casp6, which in turn cleaves Casp8, an activator of Casp3 [5] (feedback 1 in Figure 1A). In our opinion, this feedback is unlikely to account for bistable Casp3 activation via the intrinsic pathway, since Casp3 activation in response to cyto c is unaffected when the delayed Casp6 → Casp8 pathway is abrogated [5]. This conclusion is likely to hold in general, as Casp8 cleavage alone is not sufficient to stimulate its catalytic activity, but recruitment to the death-inducing signaling complex (i.e., ligand-binding to death receptors) is required [62].

It has been suggested that active Casp3 amplifies cyto c release from mitochondria by directly cleaving upstream regulators such as Bid and Bcl-2 (feedbacks 2 and 3 in Figure 1A), or by cleaving modulators of these Bcl2-family members such as Mekk1 [1]. However, the relevance of this feedback for the intrinsic pathway remains unclear, as experiments with caspase inhibitors revealed that cyto c release is caspase-independent in most cell types (e.g., [4,11,49,56,57,77]). Furthermore, the concept of Casp3-induced cyto c release is inconsistent with the fact that Casp3 activation fails in various cell types even though large amounts of cyto c were released from mitochondria (see [72] and references therein).

XIAP was shown to be cleaved by Casp3 and/or Casp8 in response to apoptotic stimulation, and such XIAP processing may result in autoamplification of Casp3 activity (feedback 4 in Figure 1A) [90,91]. In line with a predominant role of Casp8, cleavage of XIAP seems to be especially pronounced when cells are subjected to death-receptor stimulation [90,91]. By contrast, moderate [91], minor [35,36], or even no XIAP processing [92,93] was seen in response to apoptotic stimuli that initiate apoptosis via the intrinsic pathway. In addition, Casp3 may also establish a positive feedback loop by cleaving inhibitors of XIAP auto-ubiquinitation and proteasomal degradation such as PKB/Akt (feedback 4 in Figure 1A) [94,95]. Accordingly, the total XIAP abundance was shown to decrease during apoptosis (e.g., [95]), but this seems to be a cell-type–specific phenomenon, as the total amount of full-length XIAP remains essentially unchanged [91–93] or even increases [96] in other models of apoptosis.

Because of these data and due to the fact that most molecular species of the caspase cascade were shown to be continuously synthesized during apoptosis [96,97], we assumed constant total protein concentrations in our model. In order to get insight into how Casp3-mediated XIAP degradation affects the behaviour of our model, we also implemented an extended model, which takes such regulation into account (Protocol S4). Importantly, Casp3-mediated feedback cleavage of XIAP did not result in physiologically relevant bistability in a system devoid of other feedback amplification loops (Protocol S4). In addition, the qualitative conclusions drawn from Figures 2, 4, and 5 were still valid when XIAP-mediated feedback was included in the wild-type model (Figure 1B). However, these calculations also indicated that Casp3-mediated XIAP degradation may cooperate with the feedback loops discussed above, as it lowered the apoptotic threshold, A*_tot,T_, and significantly broadened the range of XIAP concentrations, where caspase activation is irreversible (BI in Figure 5).

Active Casp3 cleaves a variety of cellular substrates, and thereby initiates the execution phase of apoptosis [1]. Experimental evidence suggests that Casp3 activates multiple execution pathways in parallel and not in a sequential, cascade-like manner, since mutational inactivation of Casp3 cleavage sites abrogates specific features of apoptosis depending on the target mutated [1]. Some Casp3 substrates (e.g., PARP) are cleaved almost simultaneously with Casp3, while the processing of others (e.g., Topo I) is delayed by several hours [98,99]. Taken together, these data suggest that transient activation of the branchpoint molecule, Casp3, elicits a partial apoptotic program, which might lead to potentially harmful cellular deregulation or tissue inflammation. Active Casp3 is known to be a rather unstable protein [100], which suggests that irreversible behaviour of the caspase cascade is required to maintain Casp3 activation if upstream stimuli are removed. Experimental evidence indeed suggests that such transient stimulation occurs in living cells.

1) Cyto c release from mitochondria is thought to be a reversible as long as mitochondrial membrane potential is maintained. Because the mitochondrial membrane potential can remain unchanged long after caspases have been activated [3,4], cytosolic cyto c (i.e., the stimulus) will decline as soon as the apoptotic trigger is removed.

2) Experiments with antibodies towards the caspase-activating form of cyto c, holo–cyto c, revealed that holo–cyto c is rapidly degraded after its release into the cytosol [17].

The irreversibility mechanisms described in this paper ensure that apoptosis will fully proceed even after a decline in cyto c, and render apoptotic execution program insensitive towards survival signaling once apoptosis has been initiated. Such insensitivity is then further enhanced by delayed Casp3-mediated cleavage and thereby inactivation of various antiapoptotic signalling proteins [94].

### Proposed Experimental Verification of Bistability

Our predictions regarding all-or-none and binary integration of multiple inputs behaviour in caspase activation (Figures 2–5) can be addressed experimentally by analyzing Casp3 activation in cytosolic extracts or on a single-cell level. In cytosolic extracts, depletion and readdition experiments with various Apaf-1, Casp3, Casp9, and/or XIAP concentrations should result in all-or-none caspase activation in the BR and BI ranges in Figure 4F, but the amount of fluorescent Casp3 substrates must be chosen carefully if enzymatic activity is used as a readout. Alternatively, such multivariate analyses can be performed by microinjecting these proteins together with cyto c and/or Smac into living cells. Caspase activation can then be determined using antibodies against active Casp3 either in flow cytometric measurements or in immunofluorescence microscopy. Bistability should be confirmed by adding cyto c in combination with appropriate antagonists such as anti–cyto c antibodies, apo–cyto c, or diarylureas, which are known to inhibit apoptosome activity [18,101]. In the range of bistability, simultaneous addition of suprathreshold cyto c levels and sufficient amounts of antagonist should yield low Casp3 activity, while strong caspase activation should be observed if the antagonist is added *after* cyto c. Subsequent addition of a Casp9 inhibitor would break the feedback loops discussed in the paper, and is therefore expected to reverse Casp3 activation. The bistability measurements described above can be done on a population level (i.e., by Western blotting) if caspase activation is irreversible, but require single-cell tracking methods (e.g., real-time Casp3 assays or flow-cytometric cell sorting) in the bistable-reversible range.

### Concluding Remarks

In conclusion, we have presented a theoretical framework for quantitative experimental analyses of the intrinsic apoptosis pathway. Previous mathematical models differ from the present study in 1) the choice of apoptotic pathways, 2) the network properties focused on, and 3) the cell types analyzed. Bentele et al. [102] and Eissing et al. [50] concentrated on the extrinsic apoptosis pathway (see Figure 1A), and analyzed how switch-like behaviour arises due to stoichiometric inhibition [102] or due to positive feedback [50]. Fussenegger et al. [103] have implemented a large-scale model of both intrinsic and extrinsic pathways, and analyzed time course behaviour rather than bistability and apoptotic thresholds. Bagci et al. [104] focused on how Casp3-mediated feedback cleavage of Bcl2-family members (feedbacks 2 and 3 in Figure 1A) contributes to bistability in the intrinsic apoptosis pathway. As discussed above, these feedbacks appear to be restricted to particular cell types, where they might cooperate with those discussed here. Finally, Stucki and Simon [105] concentrated on the regulation of Casp3 degradation. The mechanisms proposed in the present paper may be combined with those discussed by Bagci et al. [104] and by Stucki and Simon [105] in order to implement more realistic models of the intrinsic apoptosis pathway.

As summarized in Protocol S5, cellular inhibitory proteins such as stoichiometric inhibitors, phosphatases, and GTPase-activating proteins frequently inhibit consecutive intermediates in cellular signaling cascades. In general, positive feedback and bistability can arise in this “shared inhibitor motif” if: 1) the signalling intermediates compete for binding to the inhibitor at least to some extent; 2) only the active form of the downstream intermediate (e.g., Casp3), but not its inactive precursor (pro-Casp3), binds to the inhibitor; and 3) the downstream intermediate (e.g., Casp3) is more abundant than the inhibitor (e.g., XIAP), which in turn needs to exceed the upstream intermediate (e.g., Casp9). As available experimental data are in accordance with these requirements, the feedback mechanism described in this paper is likely to be a widespread principle on how cells achieve ultrasensitivity, bistability, and irreversibility (Protocol S5).

## Materials and Methods

All numerical simulations were done using the MatCont Toolbox within the MATLAB (The Mathworks, Natick, Massachusetts, United States) computing environment. SBML codes of the wild-type and noncompetitive models are available in Protocols S6 and S7).

## Supporting Information

Protocol S1 Differential Equations for Wild-Type and Noncompetitive Models (115 KB PDF)

Protocol S2 XIAP-Mediated Feedback (53 KB PDF)

Protocol S3 An In Vitro Test Experiment for XIAP-Mediated Feedback (235 KB PDF)

Protocol S4 Casp3-Induced Degradation of XIAP Does Not Result in Bistability (44 KB PDF)

Protocol S5 The Shared Inhibitor Motif (72 KB PDF)

Protocol S6 SBML File for the Wild-Type Model (20 KB XML)

Protocol S7 SBML File for the Noncompetitive Model (30 KB XML)

### Accession Numbers

The Swiss-Prot (http://www.ebi.ac.uk/swissprot) accession numbers for the proteins discussed in this paper are Apaf-1 (Q4VZG8), Bax (Q07812), Bcl-2 (P10415), Bid (P55957), CD95 (P25445), c-IAP1 (Q13490), c-IAP2 (Q13489), Casp3 (P42574), Casp7 (P55210), Casp8 (Q14790), Casp9 (P55211), cyto c (P99999), Fas (P48023), NAIP (Q13075), p53 (P04637), PARP (P09874), PKB/Akt (P31749), Smac (Q9NR28), Topo I (P11387), and XIAP (P98170).

## Abbreviations

BI: bistable-irreversible
BR: bistable-reversible
Casp3: caspase-3
cyto c: cytochrome C
IAP: inhibitor of apoptosis
MB: monostable–basal activation
MG: monostable–gradual activation
MN: monostable–no activation
XIAP: X-linked IAP
