# Supplementary material for: Mathematical Modeling Identifies Inhibitors of Apoptosis as Mediators of Positive Feedback and Bistability
Source: PLoS Comput Biol. 2006 Sep 15;2(9):e120. doi: 10.1371/journal.pcbi.0020120 (PMC1570177; doi:10.1371/journal.pcbi.0020120)
Supplement: Supplementary file 1 [file pcbi.0020120.sd001.pdf]

Supplement S1: Differential Equations for Wildtype and Non-competitive Model

In the following, we provide the differential equations of the wildtype model, and explain how the wildtype model was extended in Fig. 4H and Fig. 5 in order to take non-competitive binding of Casp3 and Casp9 to XIAP into account ("Non-competitive model").

Wildtype Model

The velocities numbered according to Fig. 1B and the differential equations of the model are:

$$
\begin{aligned}
v_1 &= k_1 \cdot A^* \cdot C9 - k_{-1} \cdot A^*C9 \\
v_2 &= k_2 \cdot C3 \cdot C9 \\
v_3 &= k_3 \cdot C3 \cdot A^*C9 \\
v_4 &= k_4 \cdot C9 \cdot C3^* \\
v_5 &= k_5 \cdot A^*C9 \cdot C3^* \\
v_6 &= k_6 \cdot C3 \cdot C9^* \\
v_7 &= k_7 \cdot C3 \cdot A^*C9^* \\
v_8 &= k_8 \cdot C9^* \cdot A^* - k_{-8} \cdot A^*C9^* \\
v_9 &= k_9 \cdot C9 \cdot X - k_{-9} \cdot C9X \\
v_{10} &= k_{10} \cdot A^*C9 \cdot X - k_{-10} \cdot A^*C9X \\
v_{11} &= k_{11} \cdot C9^* \cdot X - k_{-11} \cdot C9^*X \\
v_{12} &= k_{12} \cdot A^*C9^* \cdot X - k_{-12} \cdot A^*C9^*X \\
v_{13} &= k_{13} \cdot C9X \cdot A^* - k_{-13} \cdot A^*C9X \\
v_{14} &= k_{14} \cdot C9^*X \cdot A^* - k_{-14} \cdot A^*C9^*X \\
v_{15} &= k_{15} \cdot C3^* \cdot X - k_{-15} \cdot C3^*X \\
v_{16} &= k_{-16} - k_{16} \cdot A^* \\
v_{17} &= k_{-17} - k_{17} \cdot C9 \\
v_{18} &= k_{-18} - k_{18} \cdot X \\
v_{19} &= k_{19} \cdot C9X \\
v_{20} &= k_{20} \cdot A^*C9X
\end{aligned}
$$

$$
\begin{aligned}
v_{21} &= k_{21} \cdot A^{*}C9 \\
v_{22} &= k_{-22} - k_{22} \cdot C3 \\
v_{23} &= k_{23} \cdot C3^{*} \\
v_{24} &= k_{24} \cdot C3^{*}X \\
v_{25} &= k_{25} \cdot C9^{*}X \\
v_{26} &= k_{26} \cdot C9^{*} \\
v_{27} &= k_{27} \cdot A^{*}C9^{*} \\
v_{28} &= k_{28} \cdot A^{*}C9^{*}X
\end{aligned}
$$

$$
\begin{aligned}
\frac{dA^{*}}{dt} &= -v_{1} - v_{13} - v_{8} - v_{14} + v_{16} \\
\frac{dC9}{dt} &= -v_{1} - v_{9} - v_{4} + v_{17} \\
\frac{dC9X}{dt} &= v_{9} - v_{13} - v_{19} \\
\frac{dX}{dt} &= -v_{9} - v_{10} - v_{15} - v_{11} - v_{12} + v_{18} \\
\frac{dA^{*}C9X}{dt} &= v_{10} + v_{13} - v_{20} \\
\frac{dA^{*}C9}{dt} &= v_{1} - v_{10} - v_{5} - v_{21} \\
\frac{dC3}{dt} &= -v_{2} - v_{3} - v_{6} - v_{7} + v_{22} \\
\frac{dC3^{*}}{dt} &= v_{2} + v_{3} - v_{15} + v_{6} + v_{7} - v_{23} \\
\frac{dC3^{*}X}{dt} &= v_{15} + -v_{24} \\
\frac{dC9^{*}X}{dt} &= v_{11} - v_{14} - v_{25} \\
\frac{dC9^{*}}{dt} &= v_{4} - v_{8} - v_{11} - v_{26} \\
\frac{dA^{*}C9^{*}}{dt} &= v_{5} + v_{8} - v_{12} - v_{27} \\
\frac{dA^{*}C9^{*}X}{dt} &= v_{12} + v_{14} - v_{28}
\end{aligned}
$$

Non-competitive model

XIAP-mediated feedback requires that XIAP binds caspases competitively at least to some extent, since otherwise Casp3 cannot sequester XIAP away from Casp9 (see main text). In order to analyze the role of competitive binding in more detail, we extended the wildtype model (see Fig. 1B) according to Fig. S1. Casp3, Casp9 and XIAP form a ternary complex in this 'non-competitive model' either by binding of Casp3 to Casp9-associated XIAP (reactions 33-36) or by binding of Casp9 to Casp3-associated XIAP (reactions 37-40).

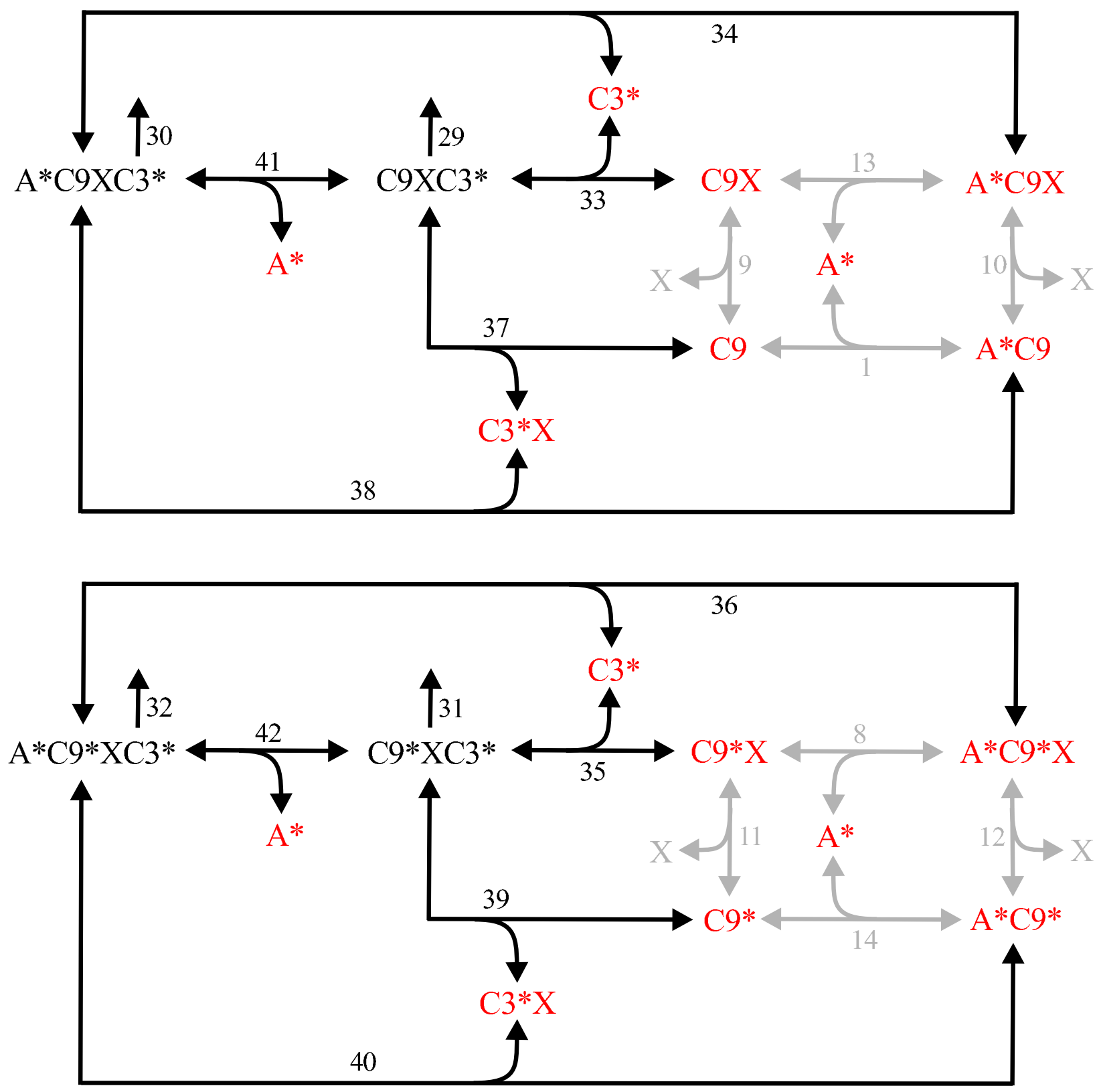

Figure S1: Model extension in the non-competitive model. The wildtype model (Fig. 1B) was extended by the black reactions (29-42). Accordingly, the differential equations of the red intermediates were altered in comparison to the wildtype model (see text), and those of the black intermediates were newly added in the non-competitive model.

While some differential equations of the wildtype model (see above) were retained in the non-competitive model, those describing the species marked in red in Fig. S1 were altered. The full model reads:

$$
\begin{aligned}
\frac{dA^*}{dt} &= -v_1 - v_{13} - v_8 - v_{14} + v_{16} - v_{41} - v_{42} \\
\frac{dC9}{dt} &= -v_1 - v_9 - v_4 + v_{17} - v_{37} \\
\frac{dC9X}{dt} &= v_9 - v_{13} - v_{19} - v_{33} \\
\frac{dX}{dt} &= -v_9 - v_{10} - v_{15} - v_{11} - v_{12} + v_{18} \\
\frac{dA^*C9X}{dt} &= v_{10} + v_{13} - v_{20} - v_{34} \\
\frac{dA^*C9}{dt} &= v_1 - v_{10} - v_5 - v_{21} - v_{38} \\
\frac{dC3}{dt} &= -v_2 - v_3 - v_6 - v_7 + v_{22} \\
\frac{dC3^*}{dt} &= v_2 + v_3 - v_{15} + v_6 + v_7 - v_{23} - v_{33} - v_{34} - v_{35} - v_{36} \\
\frac{dC3^*X}{dt} &= v_{15} + -v_{24} - v_{37} - v_{38} - v_{39} - v_{40} \\
\frac{dC9^*X}{dt} &= v_{11} - v_{14} - v_{25} - v_{35} \\
\frac{dC9^*}{dt} &= v_4 - v_8 - v_{11} - v_{26} - v_{39} \\
\frac{dA^*C9^*}{dt} &= v_5 + v_8 - v_{12} - v_{27} - v_{40} \\
\frac{dA^*C9^*X}{dt} &= v_{12} + v_{14} - v_{28} - v_{36}
\end{aligned}
$$

Additionally, four complexes between Casp3, Casp9 and XIAP were considered in the non-competitive model (black intermediates in Fig. S1). The corresponding differential equations read:

$$
\begin{aligned}
\frac{dC9XC3^*}{dt} &= -v_{29} + v_{33} + v_{37} - v_{41} \\
\frac{dA^*C9XC3^*}{dt} &= -v_{30} + v_{34} + v_{38} + v_{41}
\end{aligned}
$$

$$
\begin{aligned}
\frac{dC9^*XC3^*}{dt} &= -v_{31} + v_{35} + v_{39} - v_{42} \\
\frac{dA^*C9^*XC3^*}{dt} &= -v_{32} + v_{36} + v_{40} + v_{42}
\end{aligned}
\tag{1}
$$

The new velocities (reactions 29-42) in the non-competitive model are given by:

$$
\begin{aligned}
v_{29} &= k_{29} \cdot C9XC3^* \\
v_{30} &= k_{30} \cdot A^*C9XC3^* \\
v_{31} &= k_{31} \cdot C9^*XC3^* \\
v_{32} &= k_{32} \cdot A^*C9^*XC3^* \\
v_{33} &= k_{33} \cdot C3^* \cdot C9X - k_{-33} \cdot C9XC3^* \\
v_{34} &= k_{34} \cdot C3^* \cdot A^*C9X - k_{-34} \cdot A^*C9XC3^* \\
v_{35} &= k_{35} \cdot C3^* \cdot C9^*X - k_{-35} \cdot C9^*XC3^* \\
v_{36} &= k_{36} \cdot C3^* \cdot A^*C9^*X - k_{-36} \cdot A^*C9^*XC3^* \\
v_{37} &= k_{37} \cdot C9 \cdot C3^*X - k_{-37} \cdot C9XC3^* \\
v_{38} &= k_{38} \cdot A^*C9 \cdot C3^*X - k_{-38} \cdot A^*C9XC3^* \\
v_{39} &= k_{39} \cdot C9^* \cdot C3^*X - k_{-39} \cdot C9^*XC3^* \\
v_{40} &= k_{40} \cdot A^*C9^* \cdot C3^*X - k_{-40} \cdot A^*C9^*XC3^* \\
v_{41} &= k_{41} \cdot C9XC3^* \cdot A^* - k_{-41} \cdot A^*C9XC3^* \\
v_{42} &= k_{42} \cdot C9^*XC3^* \cdot A^* - k_{-42} \cdot A^*C9^*XC3^*
\end{aligned}
$$

The values chosen for the additional kinetic parameters in the non-competitive model are summarized in Table S1. The association and dissociation rate constants of the ternary Casp9-XIAP-Casp3 complexes (reactions 33-40 in Fig. S1) were assumed to equal those of the simple caspase-XIAP complexes, Casp3-XIAP (k15, k-15) and Casp9-XIAP (k9, k-9), and multiplied by the factors a and d (see Table S1). Thus, a and d quantify to what extend Casp3 and Casp9 bind competitively to XIAP. In Fig. 4H, both a and d were assumed to equal unity, so that competition does not occur. The competition ratio varied in Fig. 5 is given by α = a / d, and increasing caspase competition for XIAP (i.e., decreasing α) was modeled by simultaneously decreasing a and increasing d to the same extent (fold-changes).

#	Reaction	k_+	k_-	$K_D = k_- / k_+$	Notes
29	C9XC3* →	10^{-3} s^{-1}	-	-	k_+ as #16
30	A*C9XC3* →	10^{-3} s^{-1}	-	-	k_+ as #16
31	C9*XC3* →	10^{-3} s^{-1}	-	-	k_+ as #16
32	A*C9*XC3* →	10^{-3} s^{-1}	-	-	k_+ as #16
33	C9X + C3* ↔ C9XC3*	$a * k_{15}$	$d * k_{-15}$	$d / a * k_{-15} / k_{15} = 1 / \alpha * k_{-15} / k_{15}$	-
34	A*C9X + C3* ↔ A*C9XC3*	$a * k_{15}$	$d * k_{-15}$	$d / a * k_{-15} / k_{15} = 1 / \alpha * k_{-15} / k_{15}$	-
35	C9*X + C3* ↔ C9*XC3*	$a * k_{15}$	$d * k_{-15}$	$d / a * k_{-15} / k_{15} = 1 / \alpha * k_{-15} / k_{15}$	-
36	A*C9*X + C3* ↔ A*C9*XC3*	$a * k_{15}$	$d * k_{-15}$	$d / a * k_{-15} / k_{15} = 1 / \alpha * k_{-15} / k_{15}$	-
37	C9 + C3*X ↔ C9XC3*	$a * k_9$	$d * k_{-9}$	$d / a * k_{-9} / k_9 = 1 / \alpha * k_{-9} / k_9$	-
38	A*C9 + C3*X ↔ A*C9XC3*	$a * k_9$	$d * k_{-9}$	$d / a * k_{-9} / k_9 = 1 / \alpha * k_{-9} / k_9$	-
39	C9* + C3*X ↔ C9*XC3*	$a * k_9$	$d * k_{-9}$	$d / a * k_{-9} / k_9 = 1 / \alpha * k_{-9} / k_9$	-
40	A*C9* + C3*X ↔ A*C9*XC3*	$a * k_9$	$d * k_{-9}$	$d / a * k_{-9} / k_9 = 1 / \alpha * k_{-9} / k_9$	-
41	C9XC3* + A* ↔ A*C9XC3*	$2 * 10^6$ $M^{-1}s^{-1}$	0.1 s^{-1}	$5 * 10^{-8}$ M	as #1
42	C9*XC3* + A* ↔ A*C9*XC3*	$2 * 10^6$ $M^{-1}s^{-1}$	0.1 s^{-1}	$5 * 10^{-8}$ M	as #1

Table S1: Additional kinetic parameters used in the non-competitive model. The reactions numbered according to Fig. S1 (Column "#") are listed and the corresponding reactants and products are indicated (Column "Reaction"). The rate constants "k+" describe the reactions from left to right, while "k-" are the rate constants for the opposite direction (for reversible reactions). Additionally, the dissociation constants KD are indicated for reversible bimolecular reactions. The degradation rates (29-31) of the ternary complexes were set equal to those of all other molecular species in the model, and association between Casp9 and active Apaf-1 (41-42) was assumed to be unaffected by ternary complex formation (see "Notes").
