# Supplementary material for: Mathematical Modeling Identifies Inhibitors of Apoptosis as Mediators of Positive Feedback and Bistability
Source: PLoS Comput Biol. 2006 Sep 15;2(9):e120. doi: 10.1371/journal.pcbi.0020120 (PMC1570177; doi:10.1371/journal.pcbi.0020120)
Supplement: Supplementary file 2 [file pcbi.0020120.sd002.pdf]

Supplement S2: XIAP-mediated Feedback

Here, we provide detailed simulations, which show that XIAP-mediated feedback contributes to bistability and irreversibility in the wildtype model as schematically depicted in Fig. 3 of the main text.

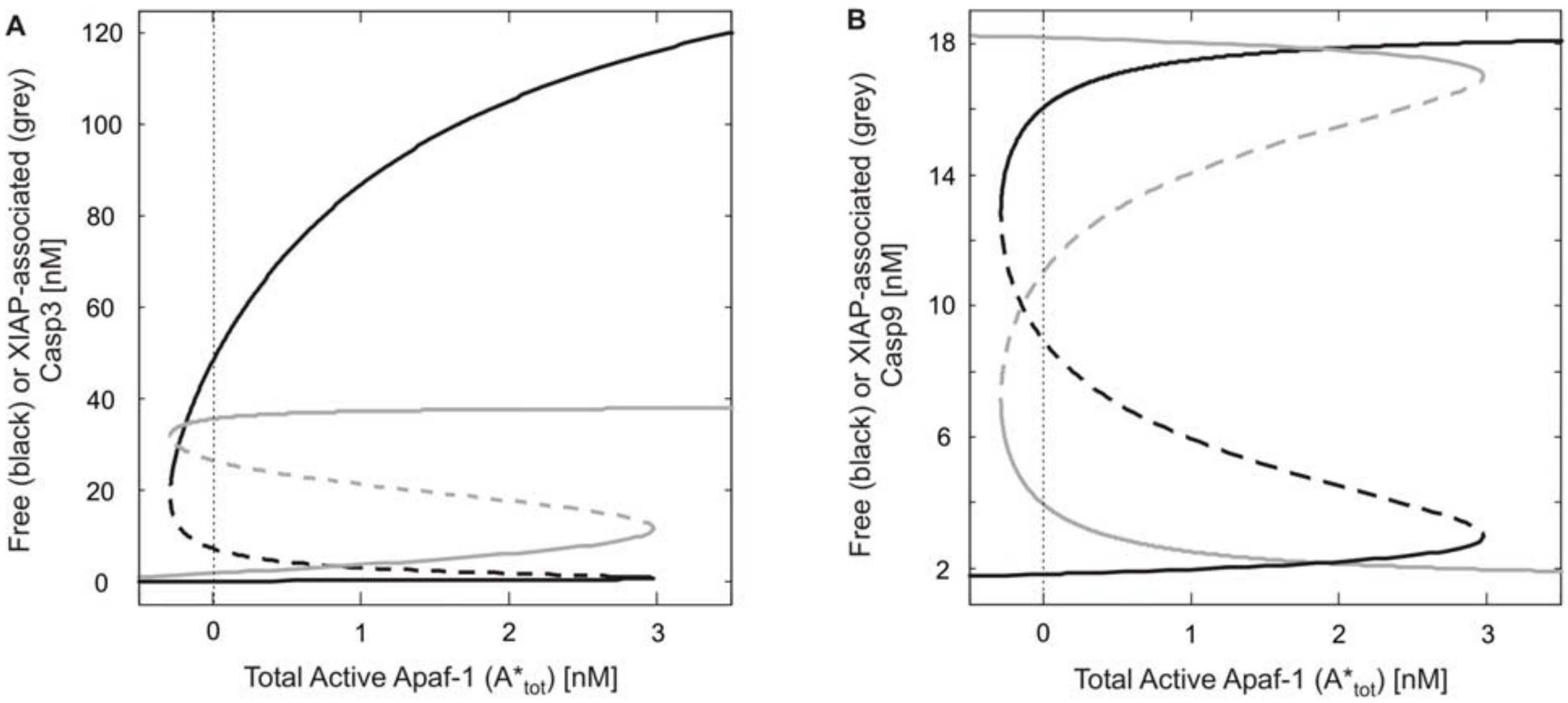

Fig. S2: XIAP-mediated Feedback. The steady state concentrations of free Casp3 (C3*; black line in A), of XIAP-associated Casp3 (C3*X; grey line in A), of free Casp9 (C9 + A*C9 + C9* + A*C9*; black line in B) and of XIAP-associated Casp9 (C9X + A*C9X + C9*X + A*C9*X; grey line in B) are plotted as a function of the stimulus, A^*_{tot}. The wildtype model (depicted in Fig. 1B) with the parameters given in Table 1 was used for simulations. The solid and dashed lines indicate stable and unstable steady states.

Upon weak stimulation the vast majority of Casp9 molecules is inhibited by excess XIAP (black and grey lines in B), so that Casp3 activation is negligible (black line in A). As the stimulus strength is increased above the threshold (~ 3 nM), XIAP is suddenly redistributed from Casp9 to Casp3 (grey lines in A and B), and Casp3 and Casp9 activities are switched on (black lines in A and B). Caspase activity is maintained even if the stimulus is removed, as Casp3, once activated, retains XIAP (grey line in A), and thereby prevents Casp9 inhibition (black line in B).
