# Supplementary material for: Mathematical Modeling Identifies Inhibitors of Apoptosis as Mediators of Positive Feedback and Bistability
Source: PLoS Comput Biol. 2006 Sep 15;2(9):e120. doi: 10.1371/journal.pcbi.0020120 (PMC1570177; doi:10.1371/journal.pcbi.0020120)
Supplement: Supplementary file 3 [file pcbi.0020120.sd003.pdf]

Supplement S3: An *in vitro* Test for XIAP-mediated Feedback

In the main text we predicted that XIAP mediates positive feedback and bistability in the intrinsic pathway. In the following we describe an *in vitro* experiment designed to confirm that sequestration of XIAP by Casp3 indeed results in feedback amplification.

	Experiment 1	Experiment 2	Control 1	Control 2
cyto c	+	+	+	+
dATP	+	+	+	+
Apaf-1	+	+	+	+
pro-Casp9 (D330A)	+	+	+	+
full-length XIAP	+	+	-	-
BIR1-BIR2 (XIAP fragment)	-	-	+	+
BIR3-RING (XIAP fragment)	-	-	+	+
pro-Casp3	-	+	-	+
Readout (Casp9 activity)	E_1	E_2	C_1	C_2

Table S2: Proposed reaction mixtures for an *in vitro* test experiment for XIAP-mediated feedback (see text)

XIAP is known to suppress the activity of apoptosome-activated Casp9 *in vitro* [1], but according to the results given in the main text excess pro-Casp3 should reverse this inhibition by sequestering XIAP away from Casp9. The proposed experiments ('Experiment 1' and 'Experiment 2') are summarized in Table S2: The pro-Casp9 mutant D330A, which is refractory towards feedback cleavage by Casp3, should be activated *in vitro* by adding cyto c, dATP, and Apaf-1 [1]. Full-length XIAP is added to both 'Experiment' reaction mixtures, while pro-Casp3 is present in Experiment 2 (feedback on), but absent in Experiment 1 (feedback on). Then, the Casp9 activities (E_1 and E_2) of both reaction mixtures should be measured as a readout.

The predicted results are shown in Fig. S3. Here, the ratio of Casp9 activities with and without Casp3 (E_2 / E_1) as a measure of (XIAP-mediated) feedback strength is plotted against the XIAP concentration. As expected, XIAP-mediated feedback is especially pronounced for intermediate XIAP concentrations, where $C9_{tot} < XIAP_{tot} < C3_{tot}$ ($C9_{tot}$ = 20 nM in these simulations). Additionally, an increase in the Casp3 concentration improves feedback strength, and also widens the range of XIAP concentrations, where feedback is observed. Therefore, the *in vitro* experiments should be done using low Casp9 concentrations, high Casp3 concentrations and intermediate XIAP concentrations ($XIAP_{tot} \approx ¾\ C3_{tot}$; see Fig. S3). Additional simulations revealed that the results shown in Fig. S3 are independent of the concentrations of active Apaf-1, but sufficiently high Apaf1-levels should be chosen in order to minimize errors.

As a control experiment, the whole procedure should be repeated with the XIAP fragments, BIR1-BIR2 (specific for Casp3) and BIR3-RING (specific for Casp9), instead of full-length XIAP (see Table S3). These controls mimick non-competitive caspase inhibition, so that the feedback strength (C_2 / C_1) is predicted to equal unity regardless of the protein concentrations chosen.

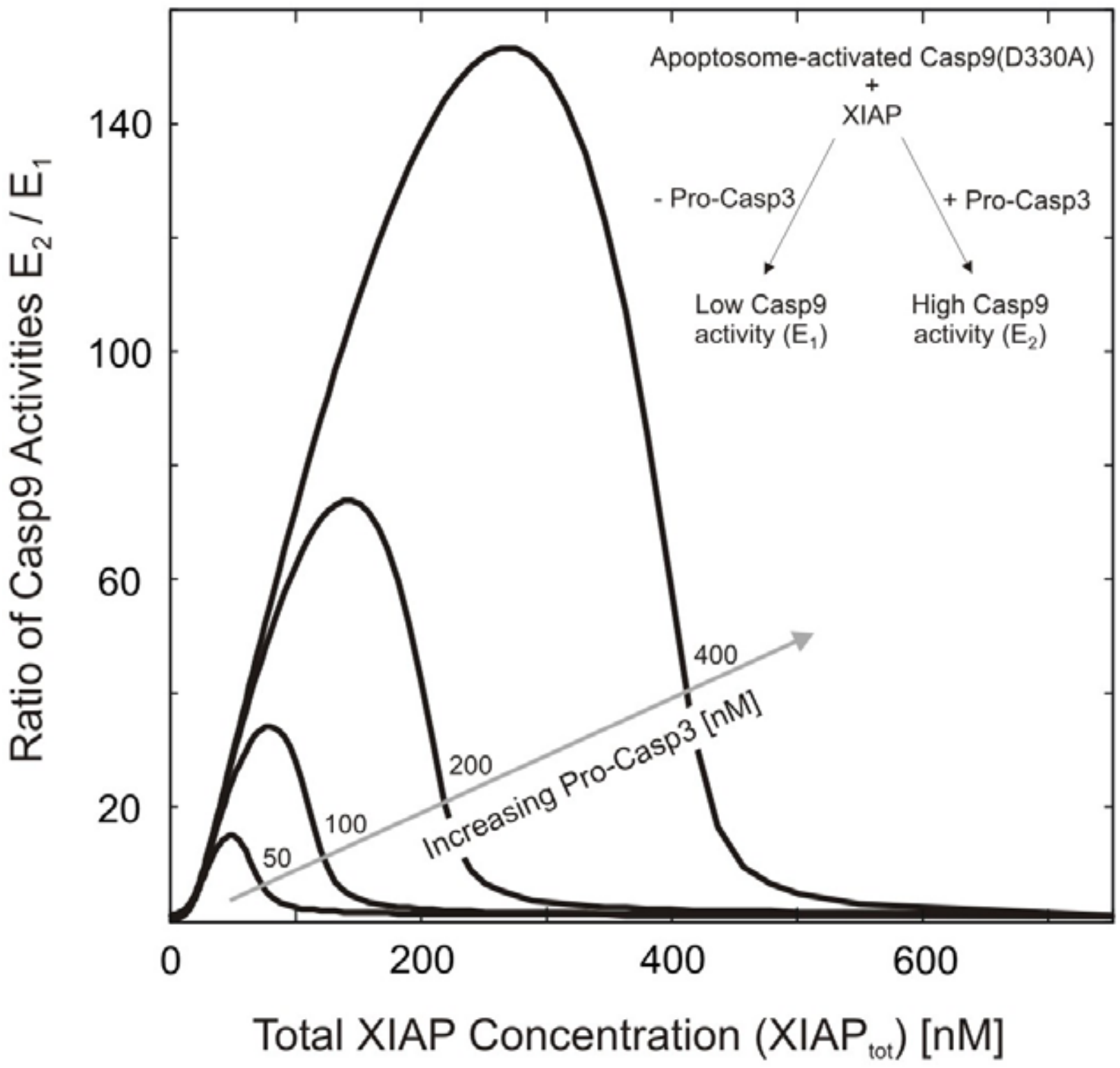

Figure S3: Predicted results of an *in vitro* test experiment for XIAP-mediated feedback. *In vitro* behaviour of the caspase cascade with mutant Casp9 (D330A) was modeled by eliminating Casp3-mediated feedback cleavage (v_4 and v_5) and protein synthesis/degradation reactions (v_{16} - v_{28}) from the differential equations given in Supplement S1 (see text for details).

REFERENCE

1. Zou H, Yang R, Hao J, Wang J, Sun C, et al. (2003) Regulation of the Apaf-1/caspase-9 apoptosome by caspase-3 and XIAP. J Biol Chem 278: 8091-8098.
