# Supplementary material for: Mathematical Modeling Identifies Inhibitors of Apoptosis as Mediators of Positive Feedback and Bistability
Source: PLoS Comput Biol. 2006 Sep 15;2(9):e120. doi: 10.1371/journal.pcbi.0020120 (PMC1570177; doi:10.1371/journal.pcbi.0020120)
Supplement: Supplementary file 4 [file pcbi.0020120.sd004.pdf]

Supplement S4: Casp3-induced Degradation of XIAP Does not Result in Bistability

Experimental evidence suggests that Casp3 activation may result in XIAP cleavage and/or degradation, although this seems to be a cell-type-specific phenomenon (see Discussion). In the following, we demonstrate that Casp3-induced XIAP degradation (‘inhibition of inhibition’ = positive circuit) does not result in physiologically relevant bistability in the absence of other feedback amplification loops.

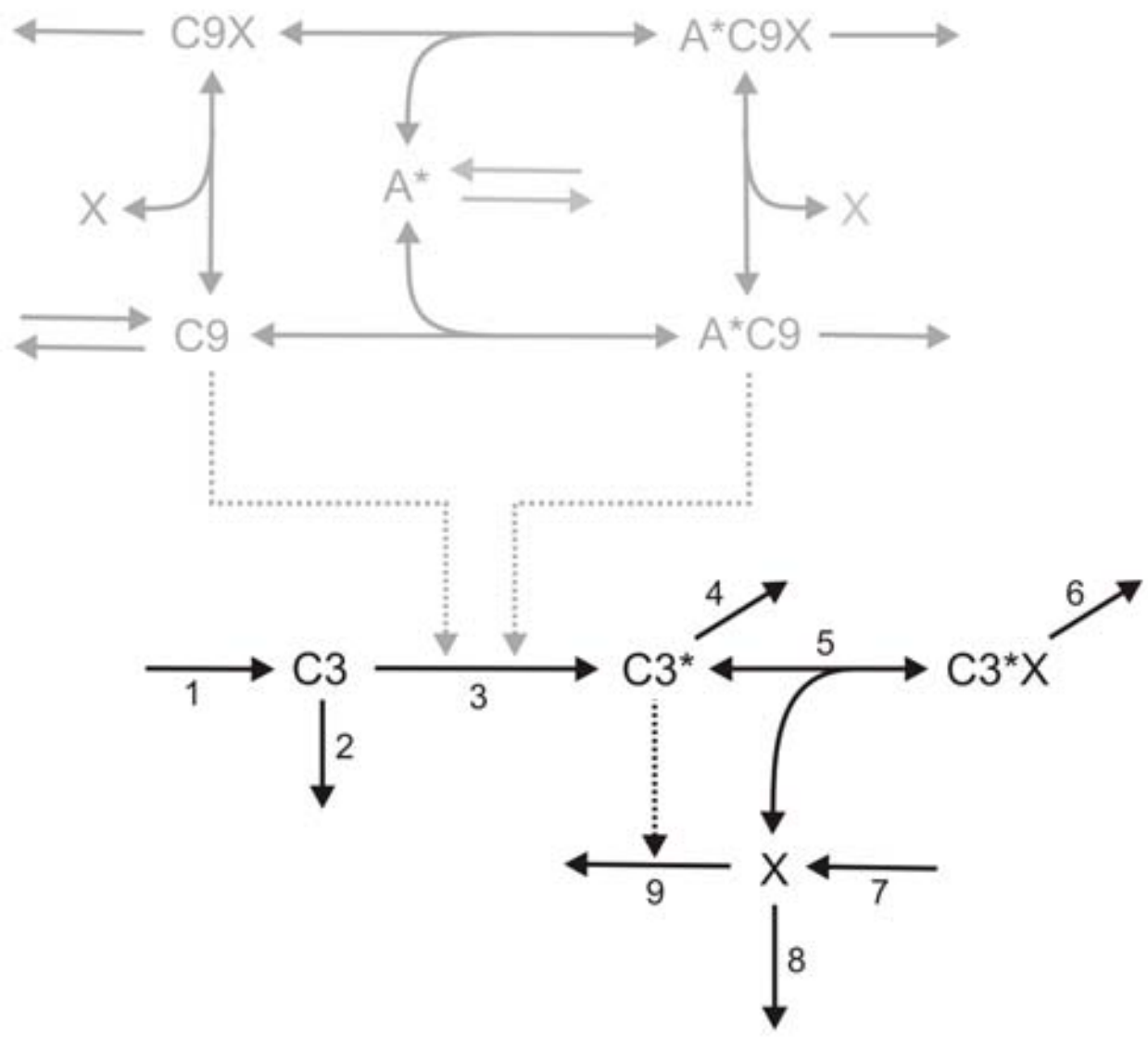

Figure S4: Model of Casp3-induced XIAP Degradation

In order to obtain analytical results, we first focussed on a simplified model of Casp3-induced XIAP degradation, which comprises only the black reactions in Fig. S4. The corresponding differential equations read:

$$\frac{dC3}{dt} = k_1 - (k_2 + k_3) \cdot C3$$

$$\frac{dC3^*}{dt} = k_3 \cdot C3 - (k_4 + k_5 \cdot X) \cdot C3^* + k_{-5} \cdot C3^*X$$

$$\frac{dC3^*X}{dt} = k_5 \cdot X \cdot C3^* - (k_{-5} + k_6) \cdot C3^*X$$

$$\frac{dX}{dt} = k_7 + k_{-5} \cdot C3^*X - (k_5 \cdot C3^* + k_8 + k_9 \cdot C3^*) \cdot X$$

The steady state condition of active Casp3 (dC3* / dt = 0) can be written as a quadratic equation in C3*, which implies that the steady state of C3* cannot be bistable.

We next numerically analyzed a more realistic model (black and grey arrows in Fig. S4), where XIAP inhibits both Casp3 and Casp9. Here, the feedback loops discussed in the paper (i.e., Casp3-mediated feedback cleavage of Casp9 and XIAP-mediated feedback) were assumed to be inactive in order to focus on the role of Casp3-mediated XIAP degradation. Hence, XIAP was assumed to bind to Casp3 and Casp9 in a non-competitive manner.

All kinetic parameters were chosen according to Tables 1 (see main text) and S1 (see Supplement S1; with $\alpha = 1$). Additionally, Casp3-mediated XIAP degradation (reaction 9 in Fig. S4) was modelled as an irreversible second-order process with the rate constant $k_9 = 3 * 10^6 M^{-1}s^{-1}$, which is the value measured for high-affinity substrates of Casp3 [1,2].

These studies revealed that Casp3-induced XIAP degradation does not result in bistability for experimentally measured protein concentrations (Casp3 = 200 nM; Casp9 = 20 nM; XIAP = 40 nM). Although some bistability could be observed for significantly different expression levels (e.g., for XIAP > 100 nM), hysteresis was restricted to a very small stimulus range, so that the stimulus-response was virtually indistinguishable from that of a monostable system.

Importantly, the physiological feedback strength (i.e., k_9 in Fig. S4) is likely to be lower than that we assumed here, as most Casp3 substrates do not match the optimal Casp3 target sequence, DEVD [1]. For example, Casp3 cleaves XIAP at a suboptimal site [3]. Likewise, Akt, a protein kinase that mediates XIAP stabilization unless it is processed and thereby inactivated by Casp3 [4], also does not contain the optimal DEVD-target sequence [5]. As high feedback strength is required for bistability [6], we can conclude that Casp3-induced XIAP degradation does not result in physiologically relevant bistability as long as other feedback amplification loops are absent

REFERENCES:

1. Margolin N, Raybuck SA, Wilson KP, Chen W, Fox T, et al. (1997) Substrate and inhibitor specificity of interleukin-1 beta-converting enzyme and related caspases. J Biol Chem 272: 7223-7228.
2. Garcia-Calvo M, Peterson EP, Rasper DM, Vaillancourt JP, Zamboni R, et al. (1999) Purification and catalytic properties of human caspase family members. Cell Death Differ 6: 362-369.
3. Deveraux QL, Leo E, Stennicke HR, Welsh K, Salvesen GS, et al. (1999) Cleavage of human inhibitor of apoptosis protein XIAP results in fragments with distinct specificities for caspases. Embo J 18: 5242-5251.
4. Dan HC, Sun M, Kaneko S, Feldman RI, Nicosia SV, et al. (2004) Akt phosphorylation and stabilization of X-linked inhibitor of apoptosis protein (XIAP). J Biol Chem 279: 5405-5412.
5. Rokudai S, Fujita N, Hashimoto Y, Tsuruo T (2000) Cleavage and inactivation of antiapoptotic Akt/PKB by caspases during apoptosis. J Cell Physiol 182: 290-296.
6. Xiong W, Ferrell JE, Jr. (2003) A positive-feedback-based bistable 'memory module' that governs a cell fate decision. Nature 426: 460-465.
