# Supplementary material for: Mathematical Modeling Identifies Inhibitors of Apoptosis as Mediators of Positive Feedback and Bistability
Source: PLoS Comput Biol. 2006 Sep 15;2(9):e120. doi: 10.1371/journal.pcbi.0020120 (PMC1570177; doi:10.1371/journal.pcbi.0020120)
Supplement: Supplementary file 5 [file pcbi.0020120.sd005.pdf]

Supplement S5: The Shared Inhibitor Motif

In the main text we showed that competitive inhibition of Casp3 and Casp9 by XIAP can bring about positive feedback and bistability in the intrinsic apoptosis pathway (see Fig. 2B, grey line; Fig. 4F). Similar conclusions regarding positive feedback and bistability also hold in general if an inhibitory protein competitively inhibits two consecutive intermediates in signal transduction cascades. This ‘shared inhibitor motif’ is schematically depicted in Fig. S5. A stimulus, S, activates the upstream intermediate, U, which then in turn catalyzes the activation of the downstream intermediate, D. Both active intermediates, U* and D*, are subject to negative regulation by the shared inhibitor, I. As indicated in Fig. S5, the shared inhibitor can either be a stoichiometric inhibitor of the intermediates (black arrows in Fig. S5) or alternatively catalyzes their deactivation, e.g., dephosphorylation (black *and* grey arrows in Fig. S5).

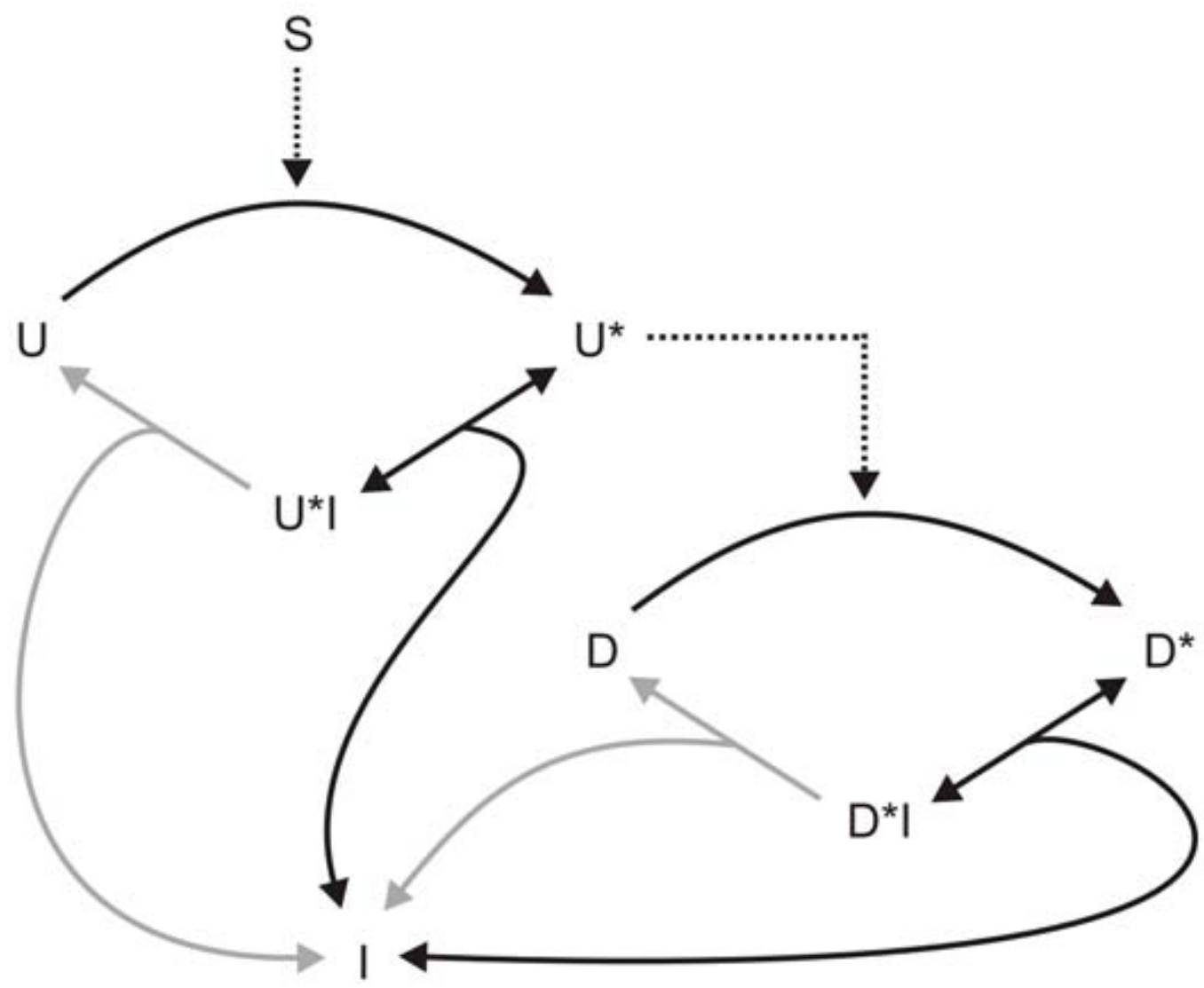

Figure S5: The Shared Inhibitor Motif

In general, bistability can arise if the shared inhibitor binds the intermediates competitively at least to some extent. Furthermore, bistability requires that *only* the active downstream intermediate, D*, but not its precursor, D, binds to the inhibitor, I. In addition to these ‘structural’ requirements, the downstream intermediate, D, needs to be significantly more abundant than the inhibitor, I, which in turn must exceed the upstream intermediate, U (see main text). Finally, the inhibitor, I, mediates particularly strong positive feedback if the downstream intermediate exceeds the dissociation constant (or the Michaelis-Menten constant) of the D*I-complex.

It should be noted that shared inhibitors, which function enzymatically (black *and* grey arrows in Fig. S5), can be efficiently sequestered by the downstream intermediate, D*, and thereby mediate positive feedback and bistability even if the D*I is only transiently formed and then broken down by catalysis (as long as the Michaelis-Menten constant is low enough).

Table S3 gives an overview on signal transduction pathways, where the ‘shared inhibitor motif’ has been reported to occur. Inhibitory proteins were subclassified into three groups, according to their biochemical mechanism of action: (i) stoichiometric inhibitors (Inh.), which reversibly sequester proteins away from their cellular targets; (ii) GTPase-activating proteins (GAP), that stimulate the intrinsic GTPase activity of small G proteins, and thereby catalyze their deactivation; (iii) Phosphatases (PP), which antagonize protein-phosphorylation cascades, are the most prominent group in Table S3.

Upstream (U)	Downstream (D)	Shared Inhibitor(s) (I)	Type	Refs.
Caspase-9	Caspase-3	X-IAP, c-IAP1/2, Survivin	Inhib.	6
Caspase-9	Caspase-7	X-IAP, c-IAP1/2, Survivin	Inhib.	6
FGFR	Mek	Sef	Inhib.	7
Grb-2	Raf	Sprouty	Inhib.	7
CyclinD / CDK4/6	Cyclin E / CDK2	p21-CIP1, p27-KIP1, p57-KIP2	Inhib.	8
Daxx (-> Ask1)	Cytochrome C	Hsp27	Inhib.	9,10
Cdc42	Rac	10 different GAPs reported	GAP	11,12
Rac	Rho	4 different GAPs reported	GAP	11,12
Cdc42	Rho	5 different GAPs reported	GAP	11,12
insulin receptor	IRS-1	LAR	PP	13
EGF receptor	HGF receptor	LAR	PP	14,15
CaMK II	AMPAR	PP1	PP	16
Aurora-B kinase	Histone 3	PP1	PP	17
Aurora-B kinase	Ndc10	PP1	PP	17
NMDA receptor	CaMK II	PP1	PP	17
p38	Caspase-3	PP2A	PP	18
Mek	Erk	PP2A	PP	19
PKA	CREB	PP2A	PP	20-22
Mek/Erk	CREB	PP2A	PP	20-22
Akt	CREB	PP2A	PP	20-22
CaMK IV	CREB	PP2A	PP	20-22
SEK1	JNK	PP2A	PP	10,22
Akt	p70S6K	PP2A	PP	21-23
PKA	Mek/Erk	PP2A	PP	21,22,24
PKCα	Mek/Erk	PP2A	PP	27,28,25
MKK6	p38	PP2C-α	PP	26
insulin receptor	IRS-1	PTP-1B	PP	13
Epo receptor	Jak2	PTP-1B	PP	27,28
Epo receptor	STAT5a/b	PTP-1B	PP	27,29
EGFR	IGF-1 receptor	PTP-1B	PP	14,30
IGF-1 receptor	EGF receptor	PTP-1B	PP	14,31
Jak2	EGF receptor	PTP-1B	PP	14,28,32
insulin receptor	Jak2	PTP-1B	PP	14,28,33,34
insulin receptor	STAT5a/b	PTP-1B	PP	14,29,33,34
PDGF receptor	Jak2	PTP-1B	PP	14,28,35
PDGF receptor	STAT5a/b	PTP-1B	PP	29,32,35
CAKβ	p130(CAS)	PTP-PEST	PP	36
CAKβ	Paxillin	PTP-PEST	PP	36
EGFreceptor	p52Shc	TCPTP	PP	37

Table S3: The ‘Shared Inhibitor motif’ is a recurrent motif in cellular signal transduction

Available experimental data suggest that bistability due to sequestration of a shared phosphatase can occur *in vivo*. Most phosphatases exhibit a single active site, i.e., they bind their substrates in a competitive manner. Additionally, they usually recognize only phosphorylated, but not non-phosphorylated, substrates, so that the structural requirements mentioned above are fulfilled. Quantitative measurements of protein abundance in the MAPK cascade revealed that the downstream intermediates in this system are (much) more abundant when compared to their upstream activators [1,2]. Finally, many phosphatases exhibit Michaelis-Menten constants in the sub-micromolar range [3,4], which suggests that strong feedback can be established (see above).

The feedback mechanism proposed in this paper may, for example, contribute to bistability in the JNK cascade [5], since PP2A was shown to dephosphorylate both JNK and its upstream activator SEK1 (see Table S3).

REFERENCES:

[1] Ferrell JE Jr.
Tripping the switch fantastic: how a protein kinase cascade can convert graded inputs into switch-like outputs.
Trends Biochem Sci. 1996

[2] Yeung K, Seitz T, Li S, Janosch P, McFerran B, Kaiser C, Fee F, Katsanakis KD, Rose DW, Mischak H, Sedivy JM, Kolch W.
Suppression of Raf-1 kinase activity and MAP kinase signalling by RKIP.
Nature. 1999 Sep 9;401(6749):173-7.

[3] Brown GC, Kholodenko BN.
Spatial gradients of cellular phospho-proteins.
FEBS Lett. 1999

[4] Zhou B, Wang ZX, Zhao Y, Brautigan DL, Zhang ZY.
The specificity of extracellular signal-regulated kinase 2 dephosphorylation by protein phosphatases.
J Biol Chem. 2002 Aug 30;277(35):31818-25.

[5] Bagowski CP, Ferrell JE Jr.
Bistability in the JNK cascade.
Curr Biol. 2001 Aug 7;11(15):1176-82.

[6] Deveraux QL, Reed JC.
IAP family proteins--suppressors of apoptosis.
Genes Dev. 1999 Feb 1;13(3):239-52.

[7] Tsang M, Dawid IB.
Promotion and attenuation of FGF signaling through the Ras-MAPK pathway.
Sci STKE. 2004 Apr 6;2004(228):pe17.

[8] Sherr CJ, Roberts JM.
CDK inhibitors: positive and negative regulators of G1-phase progression.
Genes Dev. 1999 Jun 15;13(12):1501-12.

[9] Sreedhar AS, Csermely P.
Heat shock proteins in the regulation of apoptosis: new strategies in tumor therapy: a comprehensive review.
Pharmacol Ther. 2004 Mar;101(3):227-57.

[10] Davis RJ.
Signal transduction by the JNK group of MAP kinases.
Cell. 2000 Oct 13;103(2):239-52.

[11] Burridge K, Wennerberg K.
Rho and Rac take center stage.
Cell. 2004 Jan 23;116(2):167-79.

[12] Moon SY, Zheng Y.
Rho GTPase-activating proteins in cell regulation.
Trends Cell Biol. 2003 Jan;13(1):13-22.

[13] Cheng A, Dube N, Gu F, Tremblay ML.
Coordinated action of protein tyrosine phosphatases in insulin signal transduction.
Eur J Biochem. 2002 Feb;269(4):1050-9.

[14] Ostman A, Bohmer FD.
Regulation of receptor tyrosine kinase signaling by protein tyrosine phosphatases.
Trends Cell Biol. 2001 Jun;11(6):258-66.

[15] Jo M, Stolz DB, Esplen JE, Dorko K, Michalopoulos GK, Strom SC.
Cross-talk between epidermal growth factor receptor and c-Met signal pathways in transformed cells.
J Biol Chem. 2000 Mar 24;275(12):8806-11.

[16] Hayer A, Bhalla US.
Molecular switches at the synapse emerge from receptor and kinase traffic.
PLoS Comput Biol. 2005 Jul;1(2):e20.

[17] Ceulemans H, Bollen M.
Functional diversity of protein phosphatase-1, a cellular economizer and reset button.
Physiol Rev. 2004 Jan;84(1):1-39.

[18] Alvarado-Kristensson M, Andersson T.
Protein phosphatase 2A regulates apoptosis in neutrophils by dephosphorylating both p38 MAPK and its substrate caspase 3.
J Biol Chem. 2005 Feb 18;280(7):6238-44.

[19] Saxena M, Mustelin T.
Extracellular signals and scores of phosphatases: all roads lead to MAP kinase.
Semin Immunol. 2000 Aug;12(4):387-96.

[20] Mayr B, Montminy M.
Transcriptional regulation by the phosphorylation-dependent factor CREB.
Nat Rev Mol Cell Biol. 2001 Aug;2(8):599-609.

[21] Janssens V, Goris J.
Protein phosphatase 2A: a highly regulated family of serine/threonine phosphatases implicated in cell growth and signalling.
Biochem J. 2001 Feb 1;353(Pt 3):417-39.

[22] Millward TA, Zolnierowicz S, Hemmings BA.
Regulation of protein kinase cascades by protein phosphatase 2A.
Trends Biochem Sci. 1999 May;24(5):186-91.

[23] Li Y, Corradetti MN, Inoki K, Guan KL.
TSC2: filling the GAP in the mTOR signaling pathway.
Trends Biochem Sci. 2004 Jan;29(1):32-8.

[24] Stork PJ, Schmitt JM.
Crosstalk between cAMP and MAP kinase signaling in the regulation of cell proliferation.
Trends Cell Biol. 2002 Jun;12(6):258-66.

[25] Bhalla US, Iyengar R.
Emergent properties of networks of biological signaling pathways.
Science. 1999 Jan 15;283(5400):381-7.

[26] Takekawa M, Maeda T, Saito H.
Protein phosphatase 2Calpha inhibits the human stress-responsive p38 and JNK MAPK pathways.
EMBO J. 1998 Aug 17;17(16):4744-52.

[27] Cohen J, Oren-Young L, Klingmuller U, Neumann D.
Protein tyrosine phosphatase 1B participates in the down-regulation of erythropoietin receptor signalling.
Biochem J. 2004 Jan 15;377(Pt 2):517-24.

[28] Myers MP, Andersen JN, Cheng A, Tremblay ML, Horvath CM, Parisien JP, Salmeen A, Barford D, Tonks NK.
TYK2 and JAK2 are substrates of protein-tyrosine phosphatase 1B.
J Biol Chem. 2001 Dec 21;276(51):47771-4.

[29] Aoki N, Matsuda T.
A cytosolic protein-tyrosine phosphatase PTP1B specifically dephosphorylates and deactivates prolactin-activated STAT5a and STAT5b.
J Biol Chem. 2000 Dec 15;275(50):39718-26.

[30] Hallak H, Moehren G, Tang J, Kaou M, Addas M, Hoek JB, Rubin R.
Epidermal growth factor-induced activation of the insulin-like growth factor I receptor in rat hepatocytes.
Hepatology. 2002 Dec;36(6):1509-18.

[31] Roudabush FL, Pierce KL, Maudsley S, Khan KD, Luttrell LM.
Transactivation of the EGF receptor mediates IGF-1-stimulated shc phosphorylation and ERK1/2 activation in COS-7 cells.
J Biol Chem. 2000 Jul 21;275(29):22583-9.

[32] Yamauchi T, Ueki K, Tobe K, Tamemoto H, Sekine N, Wada M, Honjo M, Takahashi M, Takahashi T, Hirai H, Tushima T, Akanuma Y, Fujita T, Komuro I, Yazaki Y, Kadowaki T.
Tyrosine phosphorylation of the EGF receptor by the kinase Jak2 is induced by growth hormone.
Nature. 1997 Nov 6;390(6655):91-6.

[33] Le MN, Kohanski RA, Wang LH, Sadowski HB.
Dual mechanism of signal transducer and activator of transcription 5 activation by the insulin receptor.
Mol Endocrinol. 2002 Dec;16(12):2764-79.

[34] Peraldi P, Filloux C, Emanuelli B, Hilton DJ, Van Obberghen E.
Insulin induces suppressor of cytokine signaling-3 tyrosine phosphorylation through janus-activated kinase.
J Biol Chem. 2001 Jul 6;276(27):24614-20.

[35] Vignais ML, Sadowski HB, Watling D, Rogers NC, Gilman M.
Platelet-derived growth factor induces phosphorylation of multiple JAK family kinases and STAT proteins.
Mol Cell Biol. 1996 Apr;16(4):1759-69.

[36] Lyons PD, Dunty JM, Schaefer EM, Schaller MD.
Inhibition of the catalytic activity of cell adhesion kinase beta by protein-tyrosine phosphatase-PEST-mediated dephosphorylation.
J Biol Chem. 2001 Jun 29;276(26):24422-31.

[37] Tiganis T, Bennett AM, Ravichandran KS, Tonks NK.
Epidermal growth factor receptor and the adaptor protein p52Shc are specific substrates of T-cell protein tyrosine phosphatase.
Mol Cell Biol. 1998 Mar;18(3):1622-34.
